# Surface Modifications of Layered Perovskite Oxysulfide Photocatalyst Y_2_Ti_2_O_5_S_2_ to Enhance Visible‐Light‐Driven Water Splitting

**DOI:** 10.1002/advs.202412326

**Published:** 2024-11-27

**Authors:** Xizhuang Liang, Junie Jhon M. Vequizo, Lihua Lin, Xiaoping Tao, Qiulian Zhu, Mamiko Nakabayashi, Daling Lu, Hiroaki Yoshida, Akira Yamakata, Takashi Hisatomi, Tsuyoshi Takata, Kazunari Domen

**Affiliations:** ^1^ Research Initiative for Supra‐Materials Interdisciplinary Cluster for Cutting Edge Research Shinshu University 4‐17‐1 Wakasato Nagano‐shi Nagano 380–8553 Japan; ^2^ School of Environmental and Material Engineering Yantai University 30 Qingquan Road Yantai 264005 China; ^3^ Institute of Engineering Innovation School of Engineering The University of Tokyo 2‐11‐16, Yayoi Bunkyo‐ku Tokyo 113–8656 Japan; ^4^ Mitsubishi Chemical Corporation Science & Innovation Center 1000 Kamoshida‐cho, Aoba‐ku Yokohama‐shi Kanagawa 227–8502 Japan; ^5^ Japan Technological Research Association of Artificial Photosynthetic Chemical Process (ARPChem) Tokyo 113–8656 Japan; ^6^ Graduate School of Natural Science and Technology Okayama University Okayama 700–8530 Japan; ^7^ Institute for Aqua Regeneration Shinshu University 4‐17‐1 Wakasato Nagano‐shi Nagano 380–8553 Japan; ^8^ Office of University Professors The University of Tokyo 2‐11‐16, Yayoi Bunkyo‐ku Tokyo 113–8656 Japan

**Keywords:** core‐shell structure, electron dynamics, overall water splitting, sequential cocatalyst decoration, Z‐scheme

## Abstract

Increasing the efficiency of visible‐light‐driven water splitting systems will require improvements in the charge separation characteristics and redox reaction kinetics associated with narrow‐bandgap photocatalysts. Although the traditional approach of loading a single cocatalyst on selective facets provides reaction sites and reduces the reaction overpotential, pronounced surface charge carrier recombination still results in limited efficiency increases. The present study demonstrates a significant improvement in the hydrogen evolution activity of the layered single‐crystal photocatalyst Y_2_Ti_2_O_5_S_2_. Increased performance is obtained through sequential loading of Pt cocatalysts using a two‐step process followed by photodeposition of Cr_2_O_3_ nanolayers. The stepwise deposition of Pt involved an impregnation‐reduction pretreatment with subsequent photodeposition and produced numerous hydrogen production sites while promoting electron capture. The Cr_2_O_3_ shells formed on Pt nanoparticles further promoted electron transfer from the Pt to the water and inhibited surface carrier recombination. Importantly, it is also possible to construct a Z‐scheme overall water splitting system using the optimized Y_2_Ti_2_O_5_S_2_ in combination with surface‐modified BiVO_4_ in the presence of [Fe(CN)_6_]^3−/4−^, yielding a solar‐to‐hydrogen energy conversion efficiency of 0.19%. This work provides insights into precise surface modifications of narrow‐bandgap photocatalysts as a means of improving the solar water splitting process.

## Graphical abstract

Fine engineering of the surface modification for single‐crystal Y_2_Ti_2_O_5_S_2_ nanosheets promotes the electron transfer, inhibits the surface carrier recombination, and enhances the H_2_ evolution activity, enabling the construction of a Z‐scheme overall water splitting system with a solar‐to‐hydrogen energy conversion efficiency of 0.19% using a long‐wavelength photoresponsive oxysulfide.

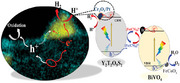

## 1 Introduction

Solar‐driven overall water splitting (OWS) using particulate photocatalysts is considered a sustainable and economical technology approach to hydrogen production.^[^ ^1^ ^]^ Even so, one‐step excitation photocatalytic OWS using visible‐light‐driven photocatalysts typically provides solar‐to‐hydrogen energy conversion (STH) efficiency values far below those required for practical applications. This lack of performance occurs due to insufficient driving of the simultaneous hydrogen evolution reaction (HER) and oxygen evolution reaction (OER) and challenges related to separating photogenerated charge carriers.^[^ ^2^ ^]^ Z‐scheme OWS, inspired by the two‐step excitation mechanism associated with photosystem I and photosystem II processes in green plants, has consequently been investigated as an alternative.^[^ ^3^ ^]^ In the latter process, the hydrogen evolution photocatalyst (HEP) and oxygen evolution photocatalyst (OEP) need only satisfy the thermodynamic requirements for the HER and OER, respectively, instead of those for both reactions simultaneously. Therefore, the Z‐scheme approach allows a greater range of materials to be considered for the construction of water splitting systems based on narrow‐bandgap photocatalysts. Even so, existing systems using sulfides, selenides, (oxy)nitrides, and dye‐sensitized photocatalysts as HEPs continue to exhibit low STH efficiencies because surface modification methods, such as cocatalyst loading, have not been fully established for these materials.^[^ ^4^ ^]^ Furthermore, even commonly used cocatalysts such as rhodium (Rh) and platinum (Pt) will capture photoexcited holes as well as electrons in some cases, leading to undesirable charge recombination at the catalyst surface.^[^ ^5^ ^]^ Recent studies have demonstrated that the site‐specific deposition of such cocatalysts is an effective means of enhancing charge separation and transfer. Li et al. observed photogenerated charge separation between different exposed crystal facets on BiVO_4_ and achieved the efficient spatial separation of electrons and holes through the photodeposition of dual cocatalysts on such facets.^[^ ^6^ ^]^ The authors previously demonstrated the spatially selective deposition of Rh/Cr_2_O_3_ and CoOOH cocatalysts on the {100} and {110} crystal facets of SrTiO_3_:Al nanocrystals. This modification led to an impressive apparent quantum yield (AQY) of over 90% under UV light and a STH efficiency of 0.65% in response to natural sunlight.^[^ ^7^ ^]^ However, this strategy involving the spatially selective capture of photogenerated charge carriers using cocatalysts is often limited to wide bandgap oxide photocatalysts. This is due to the limited availability of high‐quality non‐oxide photocatalysts having different crystal facets and of suitable cocatalysts exhibiting high activity and stability. Therefore, surface modification of narrow‐bandgap photocatalysts to promote the kinetics associated with the separation and transfer of photogenerated carriers and effectively improve the redox activity of such materials is crucial to the construction of efficient Z‐scheme OWS systems.^[^ ^8^ ^]^

Recently, Y_2_Ti_2_O_5_S_2_ (YTOS), which is a layered oxysulfide with a Ruddlesden‐Popper perovskite structure, has emerged as a promising photocatalyst for visible light‐driven water splitting. This material is composed of readily available elements, has a narrow bandgap of ≈1.9 eV that spans the HER and OER redox potentials, and exhibits exceptional physical properties. The latter include a bimolecular recombination rate constant of 1.57 × 10^−10^ cm^3^ s^−1^, an effective carrier lifetime of ≈6.14 ns, and a carrier diffusion length of ≈126 nm. YTOS also provides outstanding thermal and chemical stability compared with chalcogenides and (oxy)nitrides.^[^ ^9^ ^]^ Nevertheless, the current AQY and STH efficiency obtainable for bulk Y_2_Ti_2_O_5_S_2_ synthesized by the traditional solid‐state reaction (SSR) approach remains low because of the limited number of catalytic sites on this compound and sluggish surface catalysis.^[^ ^10^ ^]^ Nanoparticles of noble metal cocatalysts such as Rh and Pt are known to enhance the extraction of photoexcited electrons from photocatalysts, thus promoting reduction activity.^[^ ^11^ ^]^ As recently reported by Ma et al., Y_2_Ti_2_O_5_S_2_ exhibited anisotropic charge migration behavior, which was used to selectively photoload Pt@Au cocatalysts on different exposed crystal facets to improve water splitting activity.^[^ ^12^ ^]^ However, modification with such cocatalysts using conventional impregnation or photodeposition methods tends to form aggregates on the photocatalyst surface and gives minimal contact with non‐oxide photocatalysts. These effects result in an insufficient concentration of active sites and low electron transfer efficiency. Moreover, techniques for suppressing the photogenerated carrier recombination induced by these cocatalysts have not been widely studied. For all these reasons, it is important to study precise modifications of the cocatalyst surface in addition to optimizing the YTOS synthesis process and the cocatalyst deposition method.

Flux‐assisted techniques have been investigated as an alternative approach to the synthesis of oxysulfides.^[^ ^13^ ^]^ These processes reduce the reaction time that is required based on improved mass transfer efficiency in the molten salts. They also provide products having reduced levels of defects, well‐controlled particle shapes and sizes, and specific exposed crystal facets.^[^ ^14^ ^]^ In addition, a novel strategy for the stepwise deposition of cocatalysts via an impregnation‐reduction pretreatment followed by a photodeposition process has been demonstrated. This technique generates nanoparticles in intimate contact with the photocatalyst and provides abundant reaction sites, thus effectively promoting the capture of electrons.^[^ ^15^ ^]^ It has also been reported that the precise modification of the cocatalyst surface with certain inert metal oxides (such as Al_2_O_3_ and Cr_2_O_3_) can promote the separation of the photoexcited carriers participating in the catalytic reactions. This effect, in turn, increases the photocatalytic activity, although the details of the enhancement mechanism remain unclear.^[^ ^16^ ^]^

Based on such prior work, the present study prepared a YTOS single crystal nanosheet photocatalyst having minimal defects and specific exposed crystal facets using a flux‐assisted method. This material was further modified through the sequential loading of Pt cocatalysts using a two‐step process with the subsequent in situ photodeposition of Cr_2_O_3_ nanolayers. The resulting YTOS exhibited an improved AQY of 7.2% at 420 nm with regard to the photocatalytic HER in an aqueous methanol solution. A Z‐scheme OWS system incorporating the optimized YTOS with an absorption edge of 650 nm provided an AQY of 4.5% at 420 nm and an STH of 0.19%. The precise surface modification of the YTOS oxysulfide in this manner evidently provides a new path to the design and development of highly efficient solar‐powered OWS systems.

## 2 Results and Discussion

**Figure 1:**
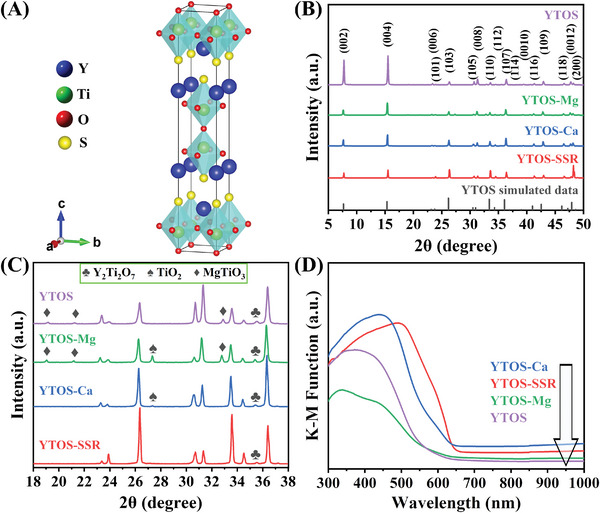
a) Unit cell structure of Y_2_Ti_2_O_5_S_2_. The blue, green, red, and yellow spheres respectively denote Y, Ti, O, and S atoms. b) Wide‐range and c) narrow‐range XRD patterns and d) UV–vis diffuse reflectance spectra for Y_2_Ti_2_O_5_S_2_ samples synthesized by different methods.

The Y_2_Ti_2_O_5_S_2_ synthesized using the flux‐assisted method with a mixture of MgCl_2_ and CaCl_2_ as the flux reagents is referred to herein as YTOS. For comparison purposes, samples were also prepared without the flux or with only CaCl_2_ or MgCl_2_ and these materials are denoted herein as YTOS‐SSR, YTOS‐Ca, and YTOS‐Mg respectively. The crystal structure and micromorphology of each specimen were initially assessed. As shown in **Figure** **1**a, the unit cell of Y_2_Ti_2_O_5_S_2_ is composed of rock‐salt [Y_2_S_2_] layers and perovskite‐related ReO_3_‐type blocks formed from −(TiO_2_)–(O)–(TiO_2_)‐ slabs (corner‐sharing [Ti_2_O_5_] octahedra) stacked in an alternating manner along the *c*‐axis. This represents a Ruddlesden‐Popper perovskite‐type layered structure belonging to the tetragonal I4/mmm space group (no. 139).^[^ ^9^, ^10^ ^]^ From the X‐ray diffraction (XRD) patterns in Figure 1b it is apparent that the ratios of the intensities of the peaks related to the {001} and {100} diffractions were 35.2, 6.8, and 12.8 in the case of the YTOS, YTOS‐Ca and YTOS‐Mg samples, respectively. These ratios were all significantly larger than the value of 1.2 determined for the YTOS‐SSR prepared using the SSR method, indicating that the crystal habit of the Y_2_Ti_2_O_5_S_2_ generated a layered compound. The use of a mixture of MgCl_2_ and CaCl_2_ as the flux evidently promoted preferential growth of the YTOS crystals along the *a*‐*b* plane, likely due to the low melting point. Figure 1c shows that all samples prepared by the flux method contained small amounts of Y_2_Ti_2_O_7_ and/or TiO_2_ as impurities. Compared with the YTOS generated using a mixture of MgCl_2_ and CaCl_2_, the YTOS‐Mg produced more intense peaks related to MgTiO_3_ as an impurity at 19.1°, 21.1°, and 32.8°.

UV–vis diffuse reflectance spectroscopy (DRS) results showed that each of the materials displayed a characteristic absorption onset at ≈650 nm. This onset is in agreement with the formation of Y_2_Ti_2_O_5_S_2_ having a corresponding bandgap energy on the order of 1.9 eV (Figure 1d; Figure S1, Supporting Information), although the absorption band position of the samples prepared by the flux‐assisted method shifted to shorter wavelengths. This can be attributed to the significant morphological effects of the layered perovskite Y_2_Ti_2_O_5_S_2_ samples. The Kubelka‐Munk (KM) function, F(*R_∞_*), represents the ratio of the absorption coefficient (*K*) to the scattering coefficient (*S*). This function can be expressed as F(*R_∞_*) = *K*/*S* = (1‐*R_∞_*)^2^/(2*R_∞_*), where *R_∞_* represents the absolute reflectance of the sample, which is generally substituted by the reflectance against a reference white plate.^[^ ^17^ ^]^ Compared with the YTOS‐SSR and YTOS‐Ca samples, the peaks in the KM profiles for the YTOS and YTOS‐Mg are at shorter wavelengths. This is ascribed to the nanosheet morphology, specifically the reduced thickness along the *c*‐axis.^[^ ^14b^ ^]^ These results are also in agreement with the ratio of the intensities of the XRD peaks corresponding to the {001} and {100} planes. The bulk YTOS‐SSR sample evidently contained some structural defects such as edge dislocations near grain boundaries (Figure S2, Supporting Information). Compared with the YTOS‐SSR, the background absorption for the samples prepared using CaCl_2_ as the flux reagent increased significantly, indicating a higher density of defects such as Ti^3+^ ions (Figure S3, Supporting Information).^[^ ^14^, ^18^ ^]^ The background absorption for the YTOS and YTOS‐Mg, while not negligible, were considerably weaker. This finding suggests that the latter contained similar defect densities due to the formation of S‐Mg‐S planar defects but at lower concentrations.^[^ ^13^ ^]^

**Figure 2:**
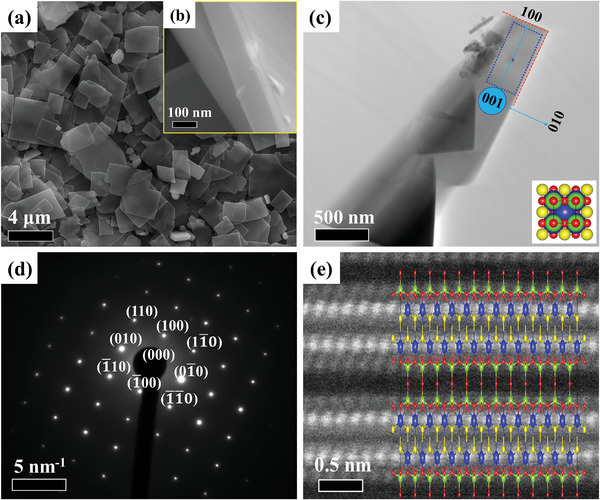
a) SEM image of YTOS sample and b) side‐view of corresponding nanocrystal sheets. c) TEM image and d) corresponding SAED pattern of YTOS sample acquired from the region within the blue rectangle. Inset: crystal structure diagram from the perspective of the (001) plane. e) Atomic‐resolution ADF‐STEM image of YTOS nanocrystal sheet viewed from side surface. The blue, green, red, and yellow spheres respectively denote Y, Ti, O, and S atoms.

Observations by scanning electron microscopy (SEM) indicated that the morphologies of the samples prepared with the flux reagents were different from that of the YTOS‐SSR. The YTOS‐SSR particles typically had truncated quadrilateral double pyramid shapes with sizes ranging from several to tens of micrometers (Figures S4a,d, Supporting Information). In contrast, some YTOS‐Ca particles exhibited plate‐like morphologies with sizes generally ranging from several hundred nanometers to a few micrometers (Figures S4b,e, Supporting Information). The YTOS sample displayed a distinct nanosheet morphology with sheet lengths ranging from several 100 nm to a few micrometers and thicknesses on the order of 120 nm, as shown in **Figure** **2**a,b. The YTOS‐Mg sample had a similar morphology to that of the YTOS but contained more MgTiO_3_ nanoparticles present as impurities (Figure S4c,f, Supporting Information). The microstructure of the YTOS nanosheets was further characterized using transmission electron microscopy (TEM), providing the images presented in Figure 2c. A selected area electron diffraction (SAED) analysis of a magnified region along the [001] direction confirmed the single crystal nature of the YTOS specimen (Figure 2d). Based on indexing the SAED pattern, the highly exposed basal planes of the nanosheets were found to comprise {001} crystal planes whereas the sides were {010} and {100} planes. Hence, the crystals grew preferentially along the *a*‐*b* plane in the presence of the flux. The insertion of molten MgCl_2_ between the layers evidently suppressed growth along the *c*‐axis, leading to the formation of the observed sheet morphology. These findings are consistent with the results obtained from the XRD and DRS analyses. High‐angle annular dark‐field‐scanning transmission electron microscopy (ADF‐STEM) images of the YTOS nanosheets demonstrated that the distribution of atoms in the specimen agreed with the cell structure of Y_2_Ti_2_O_5_S_2_ and no significant dislocations or impurity defects were visible in the observation field (Figure 2e; Figure S5, Supporting Information).

As the first step of evaluating the photocatalytic performance of these materials, the HER activity of the samples prepared by different methods was evaluated. Based on a comparison of the materials prepared without flux reagents or with only CaCl_2_ as the flux, it was found that the addition of MgCl_2_ significantly enhanced the HER activity (Figure S6, Supporting Information). This effect was attributed to the ability of MgCl_2_ to promote the formation of well‐controlled nanosheet morphologies having highly exposed {001} crystal planes (Figures S4–S7, Supporting Information). As can be seen from Figures S8 and S9 (Supporting Information), the HER activity was modified by changes in sample morphology that, in turn, resulted from changing the proportions of the flux reagents. The YTOS sample modified with Pt via the impregnation method exhibited a gradual decrease in the H_2_ evolution rate over time but demonstrated excellent HER activity in the presence of the highly alkaline sacrificial electron donors Na_2_S and Na_2_SO_3,_ providing an AQY of 10.7% at 420 nm (Figure S10, Supporting Information). Initial experimental results indicate that facet‐selective photodeposition of dual cocatalysts on faceted YTOS is not necessarily required to enhance HER activity compared to the simple impregnation method. For this reason, unless otherwise specified, Y_2_Ti_2_O_5_S_2_ synthesized using a mixture of MgCl_2_ and CaCl_2_ as the flux and subsequently loaded with Pt was used in the subsequent performance evaluation and optimization trials.

Achieving efficient Z‐scheme OWS will require the dependence on a strong electron donor to be reduced together with the activation of the YTOS photocatalyst in a neutral solution. Accordingly, methanol (MeOH) was used as the electron donor in the present work instead of the strongly alkaline compounds Na_2_S and Na_2_SO_3_. As noted, the sequential photodeposition of cocatalysts after an impregnation‐reduction pretreatment can be expected to both enhance electron capture and facilitate the formation of active sites, thereby improving photocatalytic activity.^[^ ^15^ ^]^ In particular, Pt/Cr_2_O_3_ having a core‐shell structure has emerged as a potential HER cocatalyst for water splitting. This Cr‐based modifier causes the Pt cocatalyst to selectively promote the HER and also provides a protective layer that inhibits the poisoning of the catalyst.^[^ ^16^, ^19^ ^]^ Therefore, further modifications of the YTOS surface, involving in situ photodeposition of additional Pt nanoparticles and a Cr_2_O_3_ nanolayer, were investigated in an attempt to enhance both photocatalytic HER activity and stability. The specific mechanisms responsible for the effects of such modifications on photocatalysis were analyzed.

**Figure 3:**
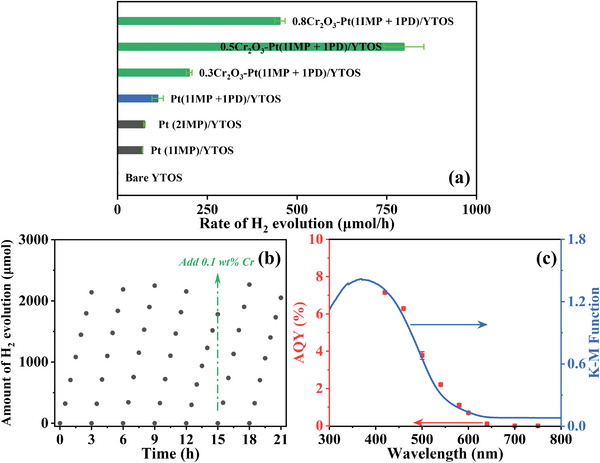
a) H_2_ evolution rates over surface‐modified YTOS samples, b) data from photocatalytic H_2_ evolution stability tests over the 0.5Cr_2_O_3_‐Pt(1IMP+1PD)/YTOS, c) AQY as a function of the incident light wavelength during the HER using 0.5Cr_2_O_3_‐Pt(1IMP+1PD)/YTOS. Reaction conditions: 200 mg of photocatalyst for (a,b) and 100 mg of photocatalyst for (c), 300 W Xe lamp equipped with a cold mirror 1 (CM 1) and with a cutoff filter L42 (λ ≥ 420 nm) for (a,b), 3 h periodic evacuation for (b), 10 vol% aqueous methanol solution, 150 mL for (a,b) and 50 mL for (c), argon background pressure of ≈7.4 kPa.

The HER activities of the surface‐modified YTOS samples in aqueous MeOH solution are summarized in **Figure** **3**a. Herein, the YTOS samples loaded with different amounts of Pt via a two‐step decoration method followed by the subsequent photodeposition of Cr_2_O_3_ are designated as *z*Cr_2_O_3_‐Pt(*x*IMP+*y*PD)/YTOS, where *x*, *y*, and *z* represent the loading amounts of Pt by impregnation‐reduction (IMP) and by photodeposition (PD) and of Cr by photodeposition with respect to the photocatalyst sample, based on mass. The YTOS specimens loaded with varying amounts of Pt via the impregnation or photodeposition methods alone are designated as Pt(*x*IMP)/YTOS or Pt(*y*PD)/YTOS, respectively. The HER activity of the Pt(2PD)/YTOS photocatalyst was extremely low. This poor performance occurred because the photodeposition of active metallic Pt nanoparticles was difficult due to the surface properties of the YTOS generated in the presence of the flux reagents (Figure S11a, Supporting Information).^[^ ^10^, ^14^ ^]^ The high‐resolution X‐ray photoelectron spectroscopy (XPS) spectrum of Pt 4*f* showed peaks at 75.7 and 72.4 eV attributed to Pt^2+^, which provided evidence for this scenario (Figure S11b, Supporting Information). Furthermore, the majority of the nanoparticles that were deposited were present in the form of large clusters and had weak interactions with the photocatalyst (Figure S11c, Supporting Information), which was not consistent with the previous report of photodeposition of Pt only on the lateral surfaces in the literature.^[^ ^12^ ^]^ The Pt(1IMP+1PD)/YTOS exhibited superior HER activity (with a product output of ≈110 µmol h^−1^) compared with the Pt(1IMP)/YTOS (≈70 µmol h^−1^) and Pt(2IMP)/YTOS (≈75 µmol h^−1^). The photocatalytic performance of the Pt(1IMP+1PD)/YTOS was significantly increased as the Cr_2_O_3_ loading was increased up to 0.5 wt.% Cr. The HER activity of ≈800 µmol h^−1^ obtained for the optimized 0.5Cr_2_O_3_‐Pt(1IMP+1PD)/YTOS sample was nearly eight times that observed prior to modification with Cr_2_O_3_.

The HER activity of the YTOS materials loaded with varying amounts of Pt via different methods, both before and after the photodeposition of Cr_2_O_3_, was further investigated. As shown in Figure S12 (Supporting Information), the presence of Cr_2_O_3_ had minimal impact on the performance of YTOS loaded with Pt through the single photodeposition method. However, this oxide significantly enhanced the activity of YTOS loaded with Pt via the single impregnation or two‐step processes. This difference can be attributed to the fact that Pt‐loaded YTOS samples exhibiting superior activity were more effective in reducing K_2_CrO_4_ at the HER sites, resulting in the formation of a Cr_2_O_3_/Pt core‐shell structure with a certain thickness.

The photocatalytic HER stability of the 0.5Cr_2_O_3_‐Pt(1IMP+1PD)/YTOS was also examined (Figure 3b). After five cycles, a 10% decrease in activity was observed but the original performance was recovered upon the addition of a minute amount of a Cr precursor solution containing 0.1 wt.% Cr. Additionally, no changes in the XRD pattern were observed after seven cycles (Figure S13, Supporting Information), suggesting that the decrease in performance was due to the partial detachment of Cr_2_O_3_ rather than deactivation. These results confirmed the exceptional chemical stability of this surface‐modified YTOS oxysulfide. The data also demonstrated that Cr_2_O_3_ played a crucial role in enhancing the photocatalytic HER activity. As can be seen from Figure 3c, the wavelength at which the photocatalytic HER was initiated was consistent with the onset of light absorption in the DRS data of YTOS, suggesting that the photoreactions were associated with bandgap transitions.^[^ ^10a^ ^]^ The AQY values for the 0.5Cr_2_O_3_‐Pt(1IMP+1PD)/YTOS at 420, 460, 500, 540, 580, 600, 640, and ≥ 700 nm were determined to be 7.2%, 6.3%, 3.8%, 2.2%, 1.1%, 0.7%, 0.1%, and 0%, respectively. These represent one of the highest AQY values yet reported for a Y_2_Ti_2_O_5_S_2_‐based photocatalyst with regard to the HER in a solution containing sacrificial agents (Table S1, Supporting Information).

**Figure 4:**
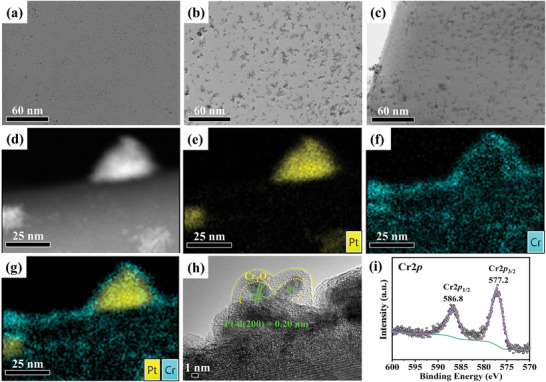
BF‐STEM images of a) Pt(1IMP)/YTOS, b) Pt(1IMP+1PD)/YTOS and c) 0.5Cr_2_O_3_‐Pt(1IMP+1PD)/YTOS specimens. d) ADF‐STEM image and e–g) corresponding STEM‐EDS maps obtained from a 0.5Cr_2_O_3_‐Pt(1IMP+1PD)/YTOS particle at a higher magnification. h) HRTEM image and i) Cr 2p XPS spectrum obtained from 0.5Cr_2_O_3_‐Pt(1IMP+1PD)/YTOS surface.

The mechanism by which the photocatalytic activity of the YTOS was enhanced following cocatalyst loading was examined by investigating the structure of the cocatalyst based on high‐resolution STEM. Bright‐field (BF)‐STEM images, ADF‐STEM images, and elemental maps acquired using STEM with energy dispersive X‐ray spectroscopy (STEM‐EDS) for a Pt(1IMP)/YTOS specimen loaded with Pt nanoparticles at a level of 1 wt.% via the impregnation technique were acquired. These showed uniform dispersion of the nanoparticles over the surfaces of the YTOS nanosheets (**Figure** **4**a; Figure S14, Supporting Information). From the high‐resolution BF‐STEM images and the corresponding particle size distribution (Figure S15a,b, Supporting Information), it was determined that the average size of the Pt(1IMP) particles was on the order of 2 nm. As the concentration of Pt loaded via the two‐step decoration method was increased to 2 wt.%, the Pt(1IMP+1PD) particles tended to merge and grow larger. This effect increased the particle size to ≈3 nm (Figure S15c,d, Supporting Information). ADF‐STEM images and STEM‐EDS elemental maps of the Pt(1IMP+1PD)/YTOS indicated that the Pt nanoparticles were partially localized in some places but, in general, were uniformly distributed over the sample surface and in close contact with the YTOS surface (Figure 4b; Figure S16, Supporting Information). Hence, it appears that the pre‐loading of Pt nuclei during the initial impregnation‐reduction step as a surface pretreatment facilitated the formation of uniformly dispersed active sites for the growth of Pt particles during the subsequent photodeposition process. This two‐step technique maintained strong interactions between the Pt and YTOS based on using the impregnation method but also took advantage of the selective deposition of active sites resulting from the photodeposition step.^[^ ^15b^ ^]^

Cr_2_O_3_ was subsequently deposited on the Pt‐loaded YTOS by photoreduction to further increase the nanoparticle size, as illustrated in Figure 4c. ADF‐STEM images and the corresponding STEM‐EDS elemental maps acquired from the 0.5Cr_2_O_3_‐Pt(1IMP+1PD)/YTOS established that Pt and Cr were present in the core and shell regions of the cocatalyst particles, respectively (Figure 4d–g). These analyses also confirmed that Cr_2_O_3_ was uniformly and selectively deposited on the surfaces of the Pt(1IMP+1PD) nanoparticles to generate a controlled core‐shell structure. STEM‐EDS elemental maps of multiple regions of the same sample led to the same conclusions, but it is worth mentioning that Cr_2_O_3_ shell layer did not completely cover all the Pt particles (Figures S17 and S18, Supporting Information). The distinct lattice fringes corresponding to a d value of 0.20 nm in Figure 4h can also be assigned to the (200) planes of Pt metal. The valence states of the 0.5Cr_2_O_3_‐Pt(1IMP+1PD)/YTOS were characterized using XPS (Figures S19 and S20, Supporting Information). Figure 4i shows doublet peaks located at 586.8 and 577.2 eV that were assigned to the Cr 2*p*_1/2_ and 2*p*_3/2_ orbitals of trivalent Cr, confirming the photodeposition of Cr_2_O_3_ on the surfaces of the metallic Pt nanoparticles.^[^ ^20^ ^]^ As discussed earlier, the HER activity was found to be dependent on the Cr_2_O_3_ loading (Figure 3a). This effect was primarily ascribed to insufficient coverage of the Pt nanoparticles at low Cr_2_O_3_ loadings such that core‐shell structures could not form (Figure S21, Supporting Information). Conversely, excessive loading resulted in a substantial increase in the thickness of the Cr_2_O_3_ nanolayers in the presence of MeOH as a sacrificial reagent. This phenomenon impeded the transfer of photogenerated carriers and increased the likelihood that photogenerated charges would undergo recombination at the surface (Figure S22, Supporting Information).^[^ ^16b^ ^]^

Transient absorption (TA) spectroscopy was used to elucidate changes in the dynamics of photogenerated charge carriers in the YTOS induced by modification with the cocatalysts. Photoexcited carriers in unmodified and cocatalyst‐loaded YTOS were monitored in the mid‐infrared (MIR) region over the time range of 3 µs–1 ms as shown in Figure S23 (Supporting Information). YTOS prepared with a mixture of MgCl_2_ and CaCl_2_ exhibited broad MIR absorption at different times when excited by 470 nm pump pulses (Figure S23a, Supporting Information). This absorption is associated with long‐lived free electrons and/or shallowly trapped electrons surviving on microsecond to millisecond timescales. The attribution of this MIR absorption to photogenerated electrons is consistent with the decay kinetics previously reported for MIR absorption in the presence of IrO_2_ and Co_3_O_4_ as OER cocatalysts loaded on YTOS for hole capture.^[^ ^21^ ^]^ Similar results were obtained in the presence of HER cocatalysts for electron capture as investigated in this work. The TA spectra obtained from the bare and Pt‐loaded YTOS also exhibited similar shapes, as can be seen in Figure S23b–d (Supporting Information), suggesting that the observed absorption signal resulted from photoexcited electrons in the YTOS photocatalyst.^[^ ^22^ ^]^

**Figure 5:**
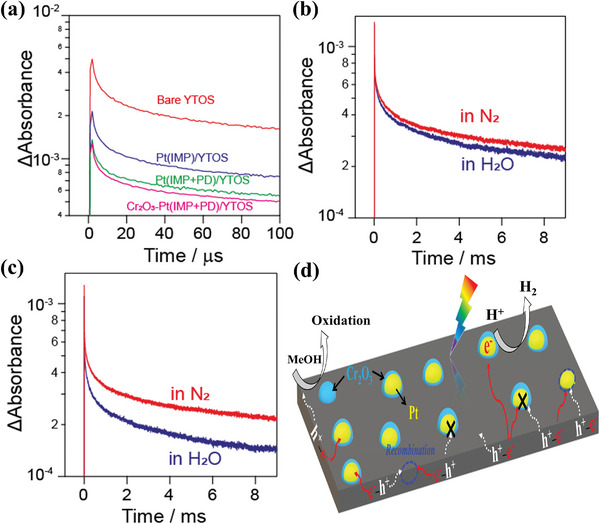
a) TA decays corresponding to electron dynamics in bare YTOS, Pt(1IMP)/YTOS, Pt(1IMP+1PD)/YTOS, and 0.5Cr_2_O_3_‐Pt(1IMP+1PD)/YTOS as probed at 2000 cm^−1^ (5000 nm, 0.25 eV) over the time span of 0–100 µs. Data showing the reactivity of electrons in b) Pt(1IMP+1PD)/YTOS and c) 0.5Cr_2_O_3_‐Pt(1IMP+1PD)/YTOS under N_2_ or water vapor at 20 Torr as probed at 2000 cm^−1^ (5000 nm, 0.25 eV) over time span of 0–9 ms. d) Schematic diagram of photodynamic processes for YTOS modified with cocatalysts in response to steady‐state excitation.

The effects of the Pt nanoparticles and Cr_2_O_3_ nanolayer modifications were further investigated by monitoring the decay profiles of photoexcited electrons in the YTOS at 5000 cm^−1^ (2000 nm, 0.62 eV) and 2000 cm^−1^ (5000 nm, 0.25 eV) on the microsecond and millisecond timescales, as illustrated in Figure S24 (Supporting Information) and **Figure** **5**a. The TA intensity for the Pt (IMP)‐loaded YTOS was found to decrease to 50% that for the bare YTOS at 100 µs after photoexcitation, whereas the intensity for the Pt(IMP+PD)‐loaded YTOS decreased to 32% of this value. After the Pt(IMP+PD)/YTOS was loaded with Cr_2_O_3_, the proportion of remaining electrons was further lowered to give a decrease of 23% (Figure 5a). This notable decrease in the TA intensity following modification with the HER cocatalysts indicates that electrons were effectively transferred to these cocatalysts. The lower TA signals subsequent to surface modifications confirmed that photoexcited electrons were rapidly transferred from the YTOS to the Pt nanoparticles. This process was enhanced by the loading of additional Pt nanoparticles and by the application of a Cr_2_O_3_ nanolayer via sequential photodeposition.

The effects of adding the Pt(IMP+PD) and of the subsequent Cr_2_O_3_ modification on the reaction kinetics during the water splitting process were further evaluated. This was done by assessing the reactivity of electrons in Pt(1IMP+1PD)/YTOS and 0.5Cr_2_O_3_‐Pt(1IMP+1PD)/YTOS samples upon exposure to water vapor based on monitoring at 5000 nm (0.25 eV). Figure 5b demonstrates that the Pt(1IMP+1PD)/YTOS generated a less intense TA decay signal and accelerated electron decay in the presence of water compared with that obtained under pure N_2_. The accelerated decay of the electrons upon exposure to water suggests that these electrons were consumed by a reduction reaction involving water.^[^ ^23^ ^]^ As shown in Figure 5c, the extent of the decrease in the TA decay signal intensity upon exposure to water was significantly greater in the trial using the 0.5Cr_2_O_3_‐Pt(1IMP+1PD)/YTOS with Cr_2_O_3_ loading. This outcome suggests that electrons were transferred more efficiently from the Pt to the water in the presence of Cr_2_O_3_. A similar trend was observed on the microsecond timescale (Figure S25, Supporting Information), implying a rapid water reduction reaction. The degree to which electron transfer from the YTOS to the HER cocatalyst was promoted by the surface modifications (Pt(IMP), Pt(IMP+PD) or Cr_2_O_3_‐Pt(IMP+PD)) was in good agreement with the enhancement of the HER activity for the corresponding samples (Figure 3a). Modification with the Pt(IMP+PD) in conjunction with Cr_2_O_3_ was evidently the most effective approach to efficient water reduction.

A representation of the photodynamic processes occurring on the YTOS after modification with cocatalysts is presented in Figure 5d. In this system, photogenerated carriers accumulated on the surface of the photocatalyst in response to steady‐state irradiation. The impregnation‐reduction method resulted in a uniform but random distribution of Pt nanoparticles, some of which permitted efficient electron transfer while others also captured holes to induce charge recombination, thus limiting effective charge carrier utilization. Evidence for this scenario is provided by the unchanged or even slightly decreased HER activity shown by the Pt(*x*IMP)/YTOS samples with increased Pt loading (*x* ≥ 2.0%, Figure 3a; Figure S10a, Supporting Information). Hence, photodeposition allowed the selective deposition of Pt nanoparticles, leading to efficient electron transfer from the YTOS to the Pt. Subsequently, Cr_2_O_3_ was photodeposited onto the pre‐loaded Pt. The Cr_2_O_3_‐coated Pt on the YTOS improved the capture of photoexcited electrons and also promoted electron injection from the Pt into the water. This effect allowed electrons to actively participate in the water reduction reaction, thereby enhancing the release of H_2_.

**Figure 6:**
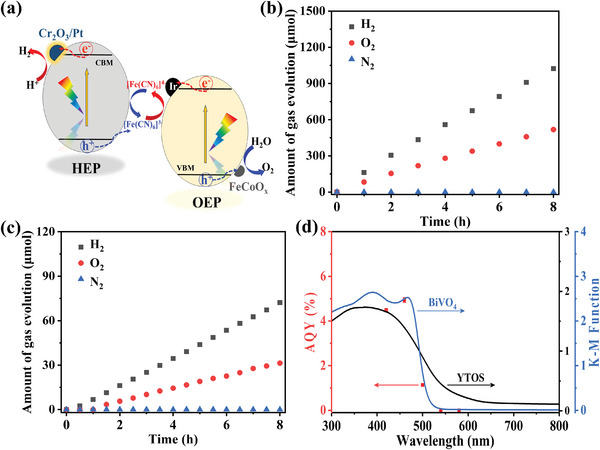
a) Energy diagram for Z‐scheme OWS system consisting of 0.5Cr_2_O_3_‐Pt(1IMP+1PD)/YTOS as the HEP, Ir‐FeCoO_x_/BiVO_4_ as the OEP and [Fe(CN)_6_]^3−/4−^ as aqueous redox mediator. Gas evolution over time during the Z‐scheme OWS reaction under b) visible light (λ ≥ 420 nm) and c) simulated sunlight (AM 1.5G, 100 mW cm^−2^). d) AQY as a function of the incident light wavelength during OWS along with the UV–vis DRS data for the HEP and the OEP. Reaction conditions: 100 mg of the HEP photocatalyst and 200 mg of the OEP photocatalyst for (b,c), 35 mg of the HEP photocatalyst and 70 mg of the OEP photocatalyst for (d), 300 W Xe lamp equipped with a cutoff filter L42 for (b) or solar simulator for (c), 5 mm [Fe(CN)_6_]^3−^, 150 mL of PBS (pH 6) for (b,c), and 50 mL of PBS (pH 6) for (d), argon at a background pressure of ≈3.7 kPa.

The present Y_2_Ti_2_O_5_S_2_ photocatalyst has a narrow bandgap and exhibits excellent HER activity and so has great potential as a component of efficient Z‐scheme OWS systems with wide spectral responses. A Z‐scheme system was thus constructed by combining the optimized 0.5Cr_2_O_3_‐Pt(1IMP+1PD)/YTOS as the HEP with surface‐modified BiVO_4_ as the OEP and [Fe(CN)_6_]^3−/4−^ as the aqueous redox mediator (**Figure** **6**a). This system produced H_2_ at 128 and O_2_ at 65 µmol h^−1^ in a stable manner and in the expected stoichiometric 2:1 molar ratio under visible light, as shown in Figure 6b. During these trials, N_2_ resulting from contamination by air was not detected, confirming that the O_2_ that was observed was generated from the water in this system. It should be noted that the Z‐scheme OWS activity was greatly affected by the concentration of [Fe(CN)_6_]^3−^ in the system (Figure S26, Supporting Information) due to reverse reactions involving the redox mediator.^[^ ^24^ ^]^ As can be seen in Figure 6c, this Z‐scheme system could also split water under simulated sunlight with a STH efficiency of 0.19%. The performance of this suspended powder Z‐scheme system was not significantly affected by the background pressure and the process maintained an STH value of 0.16% even under near‐atmospheric pressure (Figure S27, Supporting Information). As shown in Figure 6d, the optimized Z‐scheme system exhibited AQY values of 4.5%, 4.9%, and 1.1% at 420, 460, and 500 nm, respectively, during OWS. The system also showed reduced activity at wavelengths longer than 540 nm because photoexcitation of the BiVO_4_ did not occur. The present process is therefore one of the most efficient Z‐scheme water splitting systems reported to date involving a 600 nm‐class particulate photocatalyst (Table S2, Supporting Information). Comparison of water formation reaction over bare YTOS and Pt(1IMP+1PD)/YTOS under darkness was shown in Figure S28 (Supporting Information). The amounts of gaseous H_2_ and O_2_ did not change in the absence of Pt nanoparticles. However, the amounts of gaseous H_2_ and O_2_ were found to decrease gradually in the presence of Pt nanoparticles, providing evidence for the occurrence of the H_2_‐O_2_ recombination reaction on the reduction site. Moreover, our team conducted the same experiment on surface‐modified BiVO_4_, and the result showed that the amounts of gaseous H_2_ and O_2_ did not decrease, indicating that the reverse reaction did not proceed on the oxidation site.^[^ ^25^ ^]^ The future development of two‐step water splitting systems using oxysulfide‐based particulate photocatalysts will require research having several goals. These include taking advantage of the long absorption wavelengths and efficient charge separation characteristics of optimized HEPs and enhancing the activity and spectral response of potential OEPs. It would also be helpful to explore effective redox or solid‐state electron mediators.

## 3 Conclusion

Y_2_Ti_2_O_5_S_2_ single‐crystal nanosheets having minimal defects were prepared by a flux method and sequentially loaded with Pt cocatalysts through a stepwise impregnation‐reduction and photodeposition process, and then modified with Cr_2_O_3_ nanolayers. These materials exhibited remarkable photocatalytic activity during the HER in aqueous methanol solution, providing a maximum AQY of 7.2% at 420 nm. The sequential loading of Pt nanoparticles effectively promoted the transfer of photoexcited electrons from the YTOS to the cocatalysts. The selective photodeposition of Cr_2_O_3_ nanolayers on Pt cores enhanced electron transfer from the Pt to the water, such that the surface‐modified YTOS exhibited outstanding photocatalytic activity. By combining the 0.5Cr_2_O_3_‐Pt(1IMP+1PD)/YTOS photocatalyst as the HEP with surface‐modified BiVO_4_ as the OEP and [Fe(CN)_6_]^3−/4−^ as the redox mediator, a Z‐scheme system with an AQY of 4.5% at 420 nm and a STH efficiency of 0.19% during OWS was constructed. The performance of this system is among the highest yet reported for processes using narrow‐bandgap photocatalysts having absorption edge wavelengths exceeding 600 nm. This work demonstrates that the precise engineering of surface modifications can effectively promote photogenerated charge separation and redox reaction kinetics in narrow‐bandgap photocatalysts. This represents a viable approach to developing efficient artificial photosynthesis systems for water splitting.

## Conflict of Interest

The authors declare no conflict of interest.

## Supporting information



Supporting Information

## Data Availability

The data that support the findings of this study are available from the corresponding author upon reasonable request.

## References

[1] a) H. Nishiyama , T. Yamada , M. Nakabayashi , Y. Maehara , M. Yamaguchi , Y. Kuromiya , Y. Nagatsuma , H. Tokudome , S. Akiyama , T. Watanabe , R. Narushima , S. Okunaka , N. Shibata , T. Takata , T. Hisatomi , K. Domen , Nature 2021, 598, 304; 34433207 10.1038/s41586-021-03907-3

[2] X. Liang , K. Domen , Encyclopedia of Solid‐Liquid Interfaces, 1st ed, (Eds: K. Wandelt , G. Bussetti ), Elsevier, Amsterdam, Netherlands 2024, 3.

[3] a) K. Sayama , R. Yoshida , H. Kusama , K. Okabe , Y. Abe , H. Arakawa , Chem. Phys. Lett. 1997, 277, 387;

[4] a) S. Chen , Y. Qi , T. Hisatomi , Q. Ding , T. Asai , Z. Li , S. S. K. Ma , F. Zhang , K. Domen , C. Li , Angew. Chem., Int. Ed. 2015, 54, 8498; 10.1002/anie.201502686 26037473

[5] a) K. Ogawa , R. Sakamoto , C. Zhong , H. Suzuki , K. Kato , O. Tomita , K. Nakashima , A. Yamakata , T. Tachikawa , A. Saeki , H. Kageyama , R. Abe , Chem. Sci. 2022, 13, 3118; 35414879 10.1039/d1sc06054f PMC8926197

[6] a) R. Li , F. Zhang , D. Wang , J. Yang , M. Li , J. Zhu , X. Zhou , H. Han , C. Li , Nat. Commun. 2013, 4, 1432; 23385577 10.1038/ncomms2401

[7] T. Takata , J. Jiang , Y. Sakata , M. Nakabayashi , N. Shibata , V. Nandal , K. Seki , T. Hisatomi , K. Domen , Nature 2020, 581, 411. 32461647 10.1038/s41586-020-2278-9

[8] a) R. B. Chandran , S. Breen , Y. Shao , S. Ardo , A. Z. Weber , Energy Environ. Sci. 2018, 11, 115;

[9] a) G. Hyett , O. J. Rutt , Z. A. Gál , S. G. Denis , M. A. Hayward , S. J. Clarke , J. Am. Chem. Soc. 2004, 126, 1980; 14971931 10.1021/ja037763h

[10] a) Q. Wang , M. Nakabayashi , T. Hisatomi , S. Sun , S. Akiyama , Z. Wang , Z. Pan , X. Xiao , T. Watanabe , T. Yamada , N. Shibata , T. Takata , K. Domen , Nat. Mater. 2019, 18, 827; 31209390 10.1038/s41563-019-0399-z

[11] a) F. Tong , X. Liang , X. Bao , Z. Zheng , ACS Catal. 2024, 14, 11425;

[12] J. Zhang , K. Liu , B. Zhang , J. Zhang , M. Liu , Y. Xu , K. Shi , H. Wang , Z. Zhang , P. Zhou , G. Ma , J. Am. Chem. Soc. 2024, 146, 4068. 38289263 10.1021/jacs.3c12417

[13] M. Nakabayashi , K. Nishiguchi , X. Liang , T. Hisatomi , T. Takata , T. Tsuchimochi , N. Shibata , K. Domen , S. L. Ten‐no , J. Phys. Chem. C 2023, 127, 7887.

[14] a) S. K. Gupta , Y. Mao , J. Phys. Chem. C 2021, 125, 6508;

[15] a) J. Lian , D. Li , Y. Qi , N. Yang , R. Zhang , T. Xie , N. Guan , L. Li , F. Zhang , J. Energy Chem. 2021, 55, 444;

[16] a) Z. Li , R. Li , H. Jing , J. Xiao , H. Xie , F. Hong , N. Ta , X. Zhang , J. Zhu , C. Li , Nat. Catal. 2023, 6, 80;

[17] a) K. Munk , Für Tekn. Physik 1931, 12, 593;

[18] F. Zhang , K. Maeda , T. Takata , K. Domen , J. Catal. 2011, 280, 1.

[19] M. Qureshi , T. Shinagawa , N. Tsiapis , K. Takanabe , ACS Sustainable. Chem. Eng. 2017, 5, 8079.

[20] M. Yoshida , K. Takanabe , K. Maeda , A. Ishikawa , J. Kubota , Y. Sakata , Y. Ikezawa , K. Domen , J. Phys. Chem. C 2009, 113, 10151.

[21] L. Lin , V. Polliotto , J. J. M. Vequizo , X. Tao , X. Liang , Y. Ma , T. Hisatomi , T. Takata , K. Domen , ChemPhotoChem 2022, 6, 202200209.

[22] J. J. M. Vequizo , S. Nishioka , J. Hyodo , Y. Yamazaki , K. Maeda , A. Yamakata , J. Mater. Chem. A 2019, 7, 26139.

[23] a) H. Liu , M. Liu , R. Nakamura , Y. Tachibana , Appl. Catal. B: Environ. 2021, 296, 120226;

[24] a) T. Shirakawa , M. Higashi , O. Tomita , R. Abe , Sustainable Energy Fuels 2017, 1, 1065;

[25] L. Lin , Y. Ma , J. J. M. Vequizo , M. Nakabayashi , C. Gu , X. Tao , H. Yoshida , Y. Pihosh , Y. Nishina , A. Yamakata , N. Shibata , T. Hisatomi , T. Takata , K. Domen , Nat. Commun. 2024, 15, 397. 38195692 10.1038/s41467-024-44706-4 PMC10776739

