# Supplementary material for: Surface Modifications of Layered Perovskite Oxysulfide Photocatalyst Y2Ti2O5S2 to Enhance Visible‐Light‐Driven Water Splitting
Source: Adv Sci (Weinh). 2024 Nov 27;12(3):2412326. doi: 10.1002/advs.202412326 (PMC11744574; doi:10.1002/advs.202412326)
Supplement: Supplementary file 1 — Supporting Information [file ADVS-12-2412326-s001.docx]

Supporting Information

**Surface Modifications of Layered Perovskite Oxysulfide Photocatalyst Y_2_Ti_2_O_5_S_2_ to Enhance Visible-light-driven Water Splitting**

*Xizhuang Liang*, *Junie Jhon M. Vequizo*, *Lihua Lin*, *Xiaoping Tao*, *Qiulian Zhu*, *Mamiko Nakabayashi*, *Daling Lu*, *Hiroaki Yoshida*, *Akira Yamakata*, *Takashi Hisatomi*, *Tsuyoshi Takata*, *Kazunari Domen^*^*

**1. Experimental**

***1.1. Materials and reagents***

All chemicals were used without further purification in the present work. These comprised Y_2_S_3_ (99.9%, High Purity Chemical Laboratory Co., Ltd.), Y_2_O_3_ (99.99%, FUJIFLM Wako Pure Chemical Corp.), TiO_2_ (rutile, 99.99%, Rare Metallic Co., Ltd.), sulfur powder (Mesh number 75 μm pass, 99.99%, High Purity Chemical Laboratory Co., Ltd.) Bi(NO_3_)_3_·5H_2_O (99.5%, Kanto Chemical Co., Inc.), NH_4_VO_3_ (99.0%, FUJIFLM Wako Pure Chemical Corp.), H_2_PtCl_6_·6H_2_O (99.9%, FUJIFLM Wako Pure Chemical Corp.), Co(NO_3_)_2_·6H_2_O (99.95%, Kanto Chemical Co., Inc.), K_2_CrO_4_ (99.0%, FUJIFLM Wako Pure Chemical Corp.), K_3_Fe(CN)_6_ (99.0%, FUJIFLM Wako Pure Chemical Corp.), MgCl_2_ (99.99%, Sigma-Aldrich Co.), CaCl_2_ (95.0%, FUJIFLM Wako Pure Chemical Corp.), LiCl (99.0%, FUJIFLM Wako Pure Chemical Corp.), SrCl_2_ (98.0%, Kanto Chemical Co., Inc.) and BaCl_2_ (95.0%, FUJIFLM Wako Pure Chemical Corp.).

***1.2. Preparation of Y_2_Ti_2_O_5_S_2_ photocatalyst via solid-state reaction***

The Y_2_O_3_, Y_2_S_3_ and TiO_2_ serving as precursors were combined in a molar ratio of 1:2:6 and ground in a glovebox under a flow of nitrogen (>99.9995%). Sulfur powder was added to this mixture at 8 wt% to provide a sulfur-rich environment during the reaction, after which the combined materials were sealed in an evacuated quartz tube and calcined in a tube furnace. During this process, the furnace temperature was increased from room temperature to 773 K at a ramp rate of 5 K min^−1^ and then to 1073 K at a ramp rate of 1 K min^−1^, followed by holding at 1073 K for 96 h before natural cooling. After rinsing the product several times with deionized water, the raw Y_2_Ti_2_O_5_S_2_ was recovered by filtration. Residual sulfur species were removed from the product surface by annealing the as-prepared powder in air at 473 K for 1 h. Finally, the product was thoroughly rinsed with deionized water and dried under vacuum. The resultant sample is denoted herein as YTOS-SSR.

***1.3. Preparation of*** ***Y_2_Ti_2_O_5_S_2_ photocatalyst by flux-assisted method***

CaCl_2_, MgCl_2_ or a mixture of MgCl_2_ and CaCl_2_ at a molar ratio of 0.527:0.473 was used as a salt to produce the molten flux. All these flux reagents were dehydrated by calcination at 450 °C for 2 h before use. The flux was mixed with the YTOS precursor at a 5:1 mass ratio and sulfur powder was added to the YTOS precursor at 8 wt% to obtain a sulfur-rich environment. Subsequently, the mixture was sealed in an evacuated quartz tube and calcined in a tube furnace. The furnace temperature was increased from room temperature to 773 K at a ramp rate of 5 K min^−1^ and then to 1073 K at a ramp rate of 1 K min^−1^, followed by holding at 1073 K for 3 h before thermal quenching in air. After rinsing the product several times with deionized water, the raw Y_2_Ti_2_O_5_S_2_ was recovered by filtration. The as-prepared powder was subsequently annealed in air at 473 K for 1 h to remove residual sulfur species from the surface, after which the product was thoroughly rinsed with deionized water and dried under vacuum. Finally, to remove impurities and a surface amorphous layer, the powdered material was stirred in a hydrochloric acid solution (18 wt%) for 40 min, recovered by filtration, and dried under vacuum at 313 K overnight. The Y_2_Ti_2_O_5_S_2_ samples synthesized using this flux-assisted method with MgCl_2_, CaCl_2_ and a mixture of MgCl_2_ and CaCl_2_ as the flux are referred to herein as YTOS-Mg, YTOS-Ca and YTOS, respectively. YTOS specimens were also synthesized using the MgCl_2_-CaCl_2_ flux combined with the YTOS precursor at mass ratios of 0.2:1, 0.5:1, 1:1, 5:1 and 10:1. The resulting samples are referred to herein as YTOS-0.2:1, YTOS-0.5:1, YTOS-1:1, YTOS and YTOS-10:1, respectively.

Various mixed chloride molten salts were also used as fluxes. These included MgCl_2_:LiCl, MgCl_2_:SrCl_2_ and MgCl_2_:BaCl_2_ mixtures having molar ratios of 0.327:0.673, 0.464:0.536 and 0.573:0.427, respectively, combined with the Y_2_Ti_2_O_5_S_2_ precursor at a 5:1 mass ratio. Each such mixture was subsequently sealed in an evacuated quartz tube and calcined at 1073 K for 3 h followed by rapid cooling in air. The resulting materials are denoted herein as YTOS-MgLi, YTOS-MgSr and YTOS-MgBa.

***1.4. Preparation of Pt-loaded Y_2_Ti_2_O_5_S_2_ photocatalyst***

***1.4.1. Preparation of Pt-loaded Y_2_Ti_2_O_5_S_2_ photocatalyst by impregnation-reduction method***

A specific amount of an aqueous solution of H_2_PtCl_6_·6H_2_O was transferred into an evaporation dish after which 200 mg of Y_2_Ti_2_O_5_S_2_ powder was added along with 1 mL of deionized water and the mixture was stirred with a glass rod. After sonication for 1 min, the solution was completely evaporated by heating on a hot water bath. Finally, the resulting powder was heated at 573K under a flow of a mixture of N_2_ and H_2_ gases (N_2_: 100 mL min^−1^ and H_2_: 20 mL min^−1^) for 60 min. This process reduced the H_2_PtCl_6_·6H_2_O to generate metallic Pt nanoparticles on the surface of the Y_2_Ti_2_O_5_S_2_ photocatalyst. YTOS samples modified with different amounts of Pt in this manner are referred to herein as Pt(*x*IMP)/YTOS, where *x* represents the amount of Pt cocatalyst loaded in weight percent with respect to the amount of photocatalyst.

***1.4.2. Preparation of Pt-loaded Y_2_Ti_2_O_5_S_2_ photocatalyst by*** ***photodeposition method***

All photoreduction reactions were carried out in a Pyrex top-irradiation reaction vessel connected to a glass closed gas circulation system. A 200 mg quantity of Y_2_Ti_2_O_5_S_2_ powder was first dispersed in 150 mL of deionized water containing 10 vol% methanol and a specific amount of H_2_PtCl_6_·6H_2_O. After completely removing air from the reaction slurry by multiple evacuations, high-purity argon was introduced into the system to give a pressure or approximately 7.4 kPa. The suspension was subsequently irradiated with a 300 W xenon lamp equipped with a cold mirror 1 (CM1) and a cutoff filter (L42, *λ* ≥ 420 nm) for 1 h. During this process, the solution was maintained at 285 K by circulating cooling water through the system. The H_2_ gas evolved during the photodeposition was analyzed using a gas chromatograph. Following the photodeposition reaction, the Pt-loaded Y_2_Ti_2_O_5_S_2_ photocatalyst was washed and recovered by filtration. YTOS samples modified with different amounts of Pt using this technique are referred to herein as Pt(*y*PD)/YTOS, where *y* represents the amount of Pt cocatalyst loaded in weight percent with respect to the amount of photocatalyst.

***1.4.3. Preparation of Pt-loaded Y_2_Ti_2_O_5_S_2_ photocatalyst by two-step decoration method***

In some trials, Pt was loaded using a two-step method comprising a continuous combination of impregnation-reduction and photodeposition techniques. The individual processes were the same as those described above. In these experiments, a specific amount of the Pt cocatalyst was loaded onto the bare Y_2_Ti_2_O_5_S_2_ photocatalyst by impregnation followed by a H_2_-based reduction treatment. Subsequently, an additional amount of the Pt cocatalyst was loaded onto the Pt-impregnated Y_2_Ti_2_O_5_S_2_ via photodeposition. Finally, the Pt-loaded Y_2_Ti_2_O_5_S_2_ photocatalyst was washed and recovered by filtration. The YTOS samples loaded with different amounts of Pt via this two-step method are referred to herein as Pt(*x*IMP+*y*PD)/YTOS, where *x* and *y* represent the masses of Pt loaded by impregnation-reduction (IMP) and by photodeposition (PD) with respect to the mass of the photocatalyst sample.

***1.5. Preparation of*** ***Pt-loaded Y_2_Ti_2_O_5_S_2_ photocatalyst modified with Cr_2_O_3_ nanolayer***

Trials involving the photodeposition of Cr_2_O_3_ nanolayers were performed using the same closed gas circulation system as employed for the procedure described in Section 1.4.2. A 200 mg quantity of the Pt-loaded Y_2_Ti_2_O_5_S_2_ photocatalyst was first dispersed in 150 mL of deionized water containing 10 vol% methanol and a specific amount of K_2_CrO_4_ equivalent to a Cr loading of 0.3, 0.5 or 0.8 wt% with respect to the amount of photocatalyst. After completely removing air from the reaction slurry by multiple evacuations, high purity argon was introduced into the system to a pressure of 7.4 kPa. The suspension was subsequently irradiated with a 300 W xenon lamp equipped with a cold mirror 1 (CM1) for 2 h. The solution was maintained at 285 K by circulating cooling water through the system throughout the reaction and the H_2_ gas that was evolved during the photodeposition process was analyzed using a gas chromatograph. Following the photodeposition reaction, the Pt-loaded Y_2_Ti_2_O_5_S_2_ photocatalyst modified with Cr_2_O_3_ was washed and then recovered by filtration. YTOS samples loaded with different amount of Pt via the two-step method described above and then loaded with Cr_2_O_3_ in this manner are referred to herein as *z*Cr_2_O_3_-Pt(*x*IMP+*y*PD)/YTOS, where *x*, *y* and *z* represent the amounts of Pt loaded by impregnation-reduction (IMP) and by photodeposition (PD) and the quantity of Cr loaded by photodeposition, respectively, with respect to the photocatalyst sample, all based on mass.

***1.6. Preparation of monoclinic BiVO_4_ photocatalyst***

The BiVO_4_ photocatalyst was synthesized according to a previously-reported hydrothermal process.^[S1]^ In a typical procedure, 10 mmol Bi(NO_3_)_3_·5H_2_O and 10 mmol NH_4_VO_3_ were dissolved in a 2 M HNO_3_ solution, the pH of which was then slowly adjusted to approximately 0.6 by adding an aqueous ammonia solution (25-28 wt%). The resulting solution was vigorously stirred for approximately 2 h and then transferred to a Teflon-lined stainless-steel autoclave for a hydrothermal treatment at 473 K for 24 h. After natural cooling and rinsing with deionized water, the raw BiVO_4_ powder was recovered by filtration.

***1.7. Preparation of Ir-FeCoO_x_/BiVO_4_ photocatalyst***

This photocatalyst was prepared using a previously reported in situ photodeposition technique.^[S1]^ In a typical procedure, 200 mg of BiVO_4_ powder was dispersed in 50 mL of a 20 mM phosphate buffer solution (PBS, pH = 6.0) containing a specific amount of K_2_IrCl_6_ equivalent to 0.8 wt% Ir metal with respect to the mass of BiVO_4_, Co(NO_3_)_2_·6H_2_O (0.2 wt% Co metal with respect to the BiVO_4_ mass) and [Fe(CN)_6_]^3−^ at a concentration of 5 mM The suspension was thoroughly mixed and then irradiated with a 300 W xenon lamp equipped with a cold mirror (CM1) for 2 h. The resulting powder was washed and then recovered by filtration. This material is referred to herein as Ir-FeCoO*_x_*/BiVO_4_.

***1.8. Characterizations***

The structures and morphologies of the photocatalysts were characterized by powder X-ray diffraction (XRD) using a Rigaku MiniFlex 300 diffractometer with Cu-Kα radiation and by high-resolution scanning electron microscopy (SEM) using a Phenom Pharos instrument (ThermoFisher Scientific Inc.), respectively. High-resolution transmission electron microscopy (HRTEM) images, dark-field scanning TEM (ADF-STEM) images, bright-field STEM (BF-STEM) images and selected area electron diffraction (SAED) patterns were obtained using JEM-2100F and JEM-2800 instruments (JEOL). Higher-resolution observations and compositional analyses were performed using atomic-resolution STEM-EDS employing an aberration-corrected STEM instrument (JEM-ARM200F, JEOL) equipped with a silicon drift detector (EDS-SDD, DRY SD, JEOL). UV-visible diffuse reflectance spectroscopy (DRS) analyses were carried out using a V-670 spectrophotometer (JASCO Corp.) equipped with an integrating sphere. X-ray photoelectron spectroscopy (XPS) data were acquired with a PHI Quantera II spectrometer (ULVAC-PHI Inc.) employing a monochromatic Al-Kα X-ray source. The C 1*s* peak was used to calibrate the binding energies of the surface elements. Electron spin resonance (ESR) analyses were carried out using a Bruker A300 instrument (Bruker Corporation).

***1.9. Transient absorption*** ***spectroscopy***

The dynamic behavior of photoinduced charge carriers in the pristine and surface-modified YTOS photocatalysts was studied using a custom-built pump-probe system equipped with Nd:YAG lasers (Continuum, Surelite I) and custom-built spectrometers.^[S2-S4]^ Transient absorption (TA) spectra were acquired over the range from 6000 cm^-1^ (approximately 0.74 eV) to 1200 cm^-1^ (approximately 0.15 eV). In these trials, photocarriers were generated by exposing the sample to 470 nm laser pulses (duration: 6 ns, fluence: 0.1 or 1 mJ per pulse, frequency: 1 Hz). The probe light originating from a MoSi_2_ coil was focused on the sample and the transmitted infrared (IR) beam was introduced into a grating spectrometer. The monochromatic light exiting the spectrometer was quantified using a mercury cadmium telluride (MCT) detector (Kolmar). The electrical signal from this detector was amplified by an alternating current-coupled amplifier (Stanford Research Systems, SR560, 1 MHz) capable of tracking responses on time scales from one microsecond to several milliseconds. The decay profiles for the transient absorptions at 5000 cm^-1^ (2000 nm, approximately 0.62 eV) and 2000 cm^−1^ (5000 nm, approximately 0.25 eV) on the microsecond and millisecond timescales were assessed to investigate the decay behavior of photoinduced electrons. The film specimens required for these analyses were prepared by homogeneously dispersing a portion of each photocatalyst powder in water followed by the drop-casting of the resulting suspension onto a CaF_2_ substrate. Each film/CaF_2_ specimen was placed in a stainless-steel reaction cell that was subsequently evacuated to a pressure on the order of 10^-5^ Torr. Laser pulses (470 nm, 1 mJ per pulse) were used to excite the pristine and surface-modified YTOS photocatalysts via bandgap transitions. Data were recorded at room temperature under N_2_ gas or water vapor, both at a pressure of 20 Torr.

***1.10.*** ***Photocatalytic water splitting*** ***experiments***

***1.10.1.*** ***H_2_ evolution reaction***

All trials were carried out in a Pyrex top-irradiation reaction vessel connected to a glass closed gas circulation system. In trials involving the H_2_ evolution half reaction, 200 mg of the photocatalyst was dispersed in 150 mL of deionized water containing 10 vol% methanol or 20 mM Na_2_S-Na_2_SO_3_ as a sacrificial electron donor. After completely removing air from the reaction slurry by multiple evacuations, high-purity argon was introduced into the system to a pressure of approximately 7.4 kPa prior to the reaction. The suspension was then irradiated using a 300 W xenon lamp equipped with a cold mirror 1 (CM1) and a cutoff filter L42 (*λ* ≥ 420 nm). The solution was maintained at 285 K by circulating cooling water through the system during the reaction. The gaseous products were analyzed at 30 min intervals using a gas chromatograph (Shimadzu Corp.) equipped with a thermal conductivity detector and 5Å molecular sieve columns, employing argon (>99.999%) as the carrier gas.

***1.10.2.*** ***Z-scheme overall water splitting reactions***

The Z-scheme overall water splitting trials were performed in the same closed gas circulation system following the same procedure as described for the H_2_ evolution half reaction in the preceding section, except that sacrificial electron donors were not used. The hydrogen evolution photocatalyst (HEP) and oxygen evolution photocatalyst (OEP) were dispersed in 150 mL of 20 mM PBS (pH = 6.0) containing 5 mM K_3_[Fe(CN)_6_]. After completely removing air from the reaction slurry by multiple evacuations, high purity argon was introduced into the system to a pressure of approximately 3.7, 50 or 90 kPa prior to the reaction. The suspension was then irradiated using either a 300 W xenon lamp equipped with a cold mirror 1 (CM1) and a cutoff filter L42 (*λ* ≥ 420 nm) or a solar simulator (AM 1.5G, 100 mW cm^−2^). The reactant solution was maintained at 285 K by circulating cooling water through the system during the reaction. Achieving balanced concentrations of the [Fe(CN)_6_]^3-/4-^ ions acting as aqueous redox mediators required the reaction system to be exposed to light prior to the experiment. Following evacuation, the evolved gaseous products were analyzed at 30 min intervals using a gas chromatograph (Shimadzu Corp.) equipped with a thermal conductivity detector and 5Å molecular sieve columns, employing argon (>99.999%) as the carrier gas.

***1.10.3. Estimation of AQY and STH efficiency***

The apparent quantum yield (AQY), also known as the apparent quantum efficiency (AQE) or external quantum efficiency (EQE), is one of the key factors used to evaluate photocatalytic performance. This value is defined as the number of electrons that participate in the water-splitting reaction divided by the number of incident photons at a specific wavelength. The solar-to-hydrogen energy conversion efficiency (STH efficiency) is another important parameter used to compare the overall water splitting performance of photocatalysts. Both the AQY and STH are directly proportional to the gas evolution activity of the photocatalyst. The AQY can be expressed as

$$AQY=\frac{number of reacted electrons}{number of incident photons}=\frac{n\times N(H_{2} or O_{2})}{N_{inc}}\times100\% Eq.\left( 1 \right),$$

where *n* is the number of electrons/holes consumed in the formation of one H_2_/O_2_ molecule, $N(H_{2} or O_{2})$is the quantity of H_2_ or O_2_ molecules generated during a given time interval, and $N_{inc}$ is the number of incident photons reaching the reaction solution during the same time interval. In the case of H_2_ evolution from a solution containing a sacrificial reagent, *n* will equal 2. For H_2_ and O_2_ evolution from a Z-scheme water splitting system based on two-step photoexcitation, *n* will equal 4 and 8, respectively. The STH efficiency is calculated as

$$\mathrm{STH}\mathrm{efficiency}=\frac{Output energy as H_{2}}{Energy of incident solar light}=\frac{R_{H_{2}}\times\Delta G}{P_{\mathrm{su}n}\times S_{\mathrm{area}}}\times100\% Eq.\left( 2 \right),$$

where $R_{H_{2}}$ is the rate of H_2_ evolution, $P_{sun}$ is the energy flux of the simulated sunlight applied to the system (AM 1.5G, 100 mW cm^-2^), $S_{area}$ is the geometrical area of the reactor that is irradiated and *ΔG* (237.2 kJ mol^-1^) is the change in the Gibbs free energy associated with the water splitting reaction.


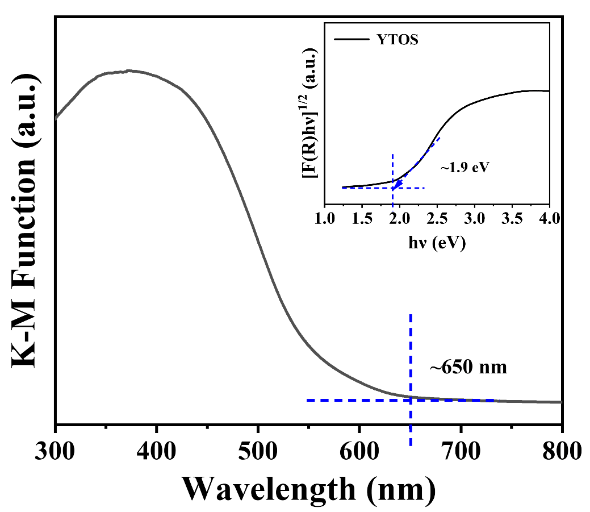


**Figure S1.** UV-visible DRS data for Y_2_Ti_2_O_5_S_2_ sample prepared using a flux composed of MgCl_2_ and CaCl_2_. Inset: a Tauc plot of [*F*(*R*)*hν*]^1/2^ vs. *hν* for the same Y_2_Ti_2_O_5_S_2_ sample, where *F*(*R*) is the Kubelka–Munk function.

**
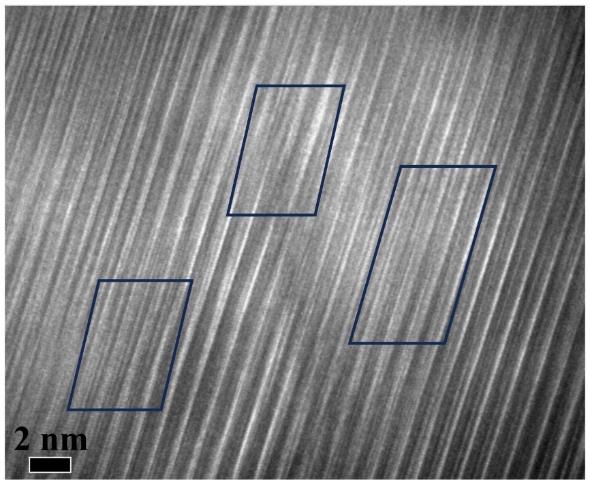
**

**Figure S2.** HRTEM image of YTOS-SSR specimen. Obvious dislocation defects are indicated by the blue parallelograms.

**

**

**Figure S3.** ESR spectra of YTOS-SSR and YTOS-Ca. Experimental conditions: dark; room temperature.

As shown in Figure S3, the electron spin resonance (ESR) was performed to study Ti^3+^ defects in YTOS-SSR and YTOS-Ca samples. In the case of YTOS-Ca sample, the signals for Ti^3+^ were observed at 3591 and 3661 G (g=1.96 and 1.92, respectively).^[S5,S6]^ Compared with the YTOS-SSR, the signal intensity increased with the introduction of CaCl_2_ as the flux reagent.


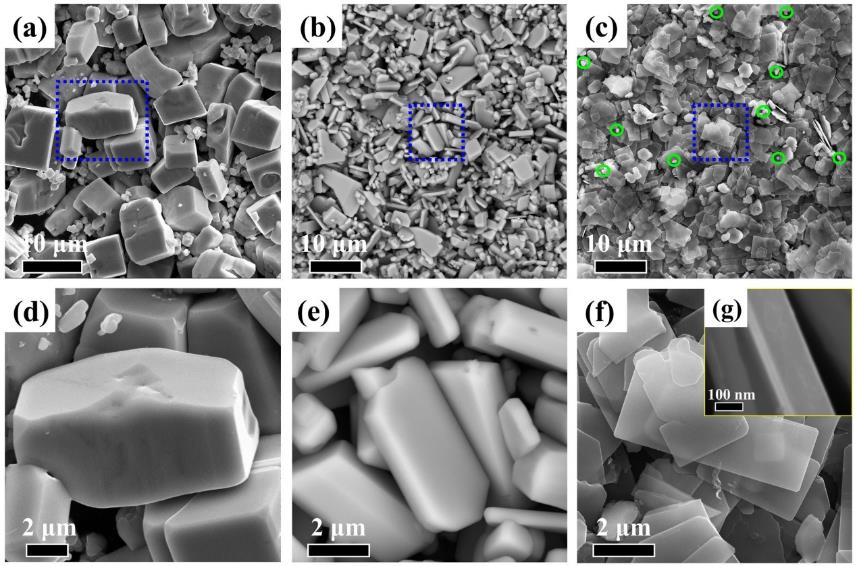


**Figure S4.** (a-c) SEM images of YTOS samples and (d-f) high-resolution SEM images of the regions indicated by blue squares. The specimens comprise (a, d) YTOS-SSR, (b, e) YTOS-Ca, and (c, f) YTOS-Mg. In (c), MgTiO_3_ particles present as impurities are indicated by green circles. Inset: (g) A side-view of the YTOS-Mg nanosheet.


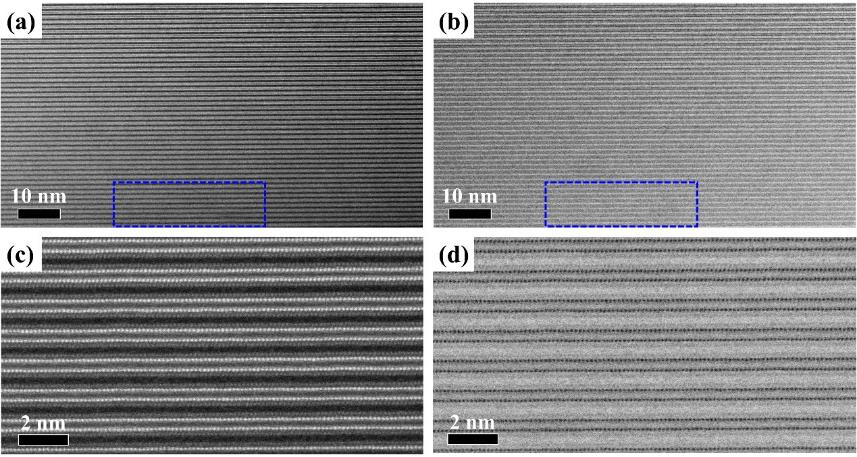


**Figure S5.** (a, c) Atomic resolution ADF-STEM images and (b, d) corresponding BF-STEM images of YTOS crystallite viewed from side surface.

**
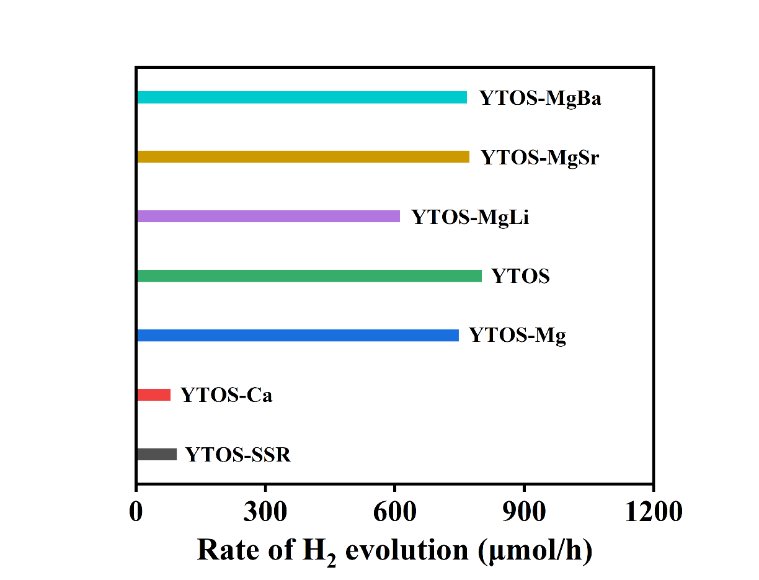
**

**Figure S6.** H_2_ evolution rates over Pt-modified Y_2_Ti_2_O_5_S_2_ samples prepared with different flux reagents. Reaction conditions: 200 mg photocatalyst loaded with 1 wt% Pt via the impregnation method, 300 W Xe lamp equipped with a cold mirror 1 (CM 1) and a cutoff filter L42 (*λ* ≥ 420 nm) as the light source, 20 mM Na_2_S-Na_2_SO_3_ solution, 150 mL water, argon at a background pressure of approximately 7.4 kPa.


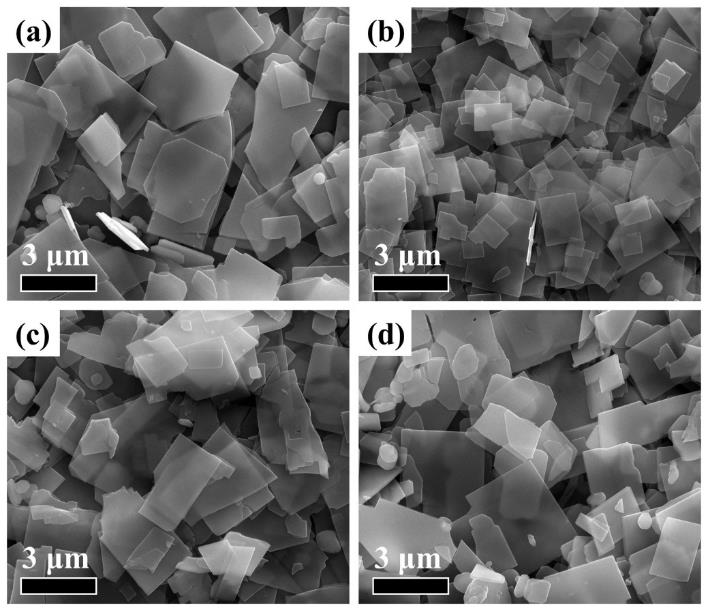


**Figure S7.** SEM images of as-prepared (a) YTOS, (b) YTOS-MgLi, (c) YTOS-MgSr, and (d) YTOS-MgBa.


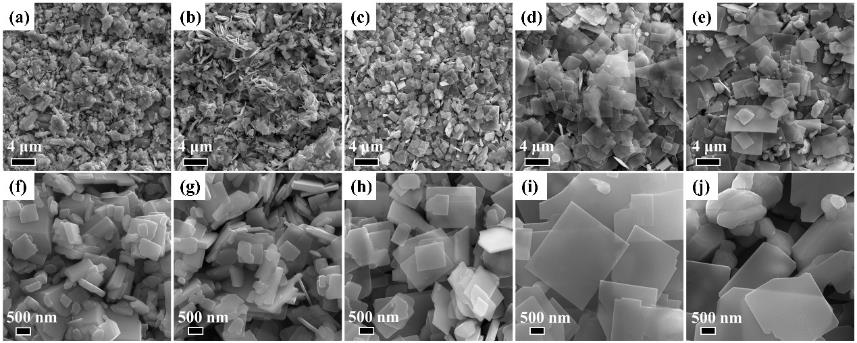


**Figure S8.** SEM images of as-prepared (a, f) YTOS-0.2:1, (b, g) YTOS-0.5:1, (c, h) YTOS-1:1, (d, i) YTOS, and (e, j) YTOS-10:1.

As the relative amount of the flux was increased during the synthesis process, the bulk particles initially became thinner (Figure S8) after which nanosheets with exposed (001) crystal facets were formed, consistent with the analysis provided in the main paper.


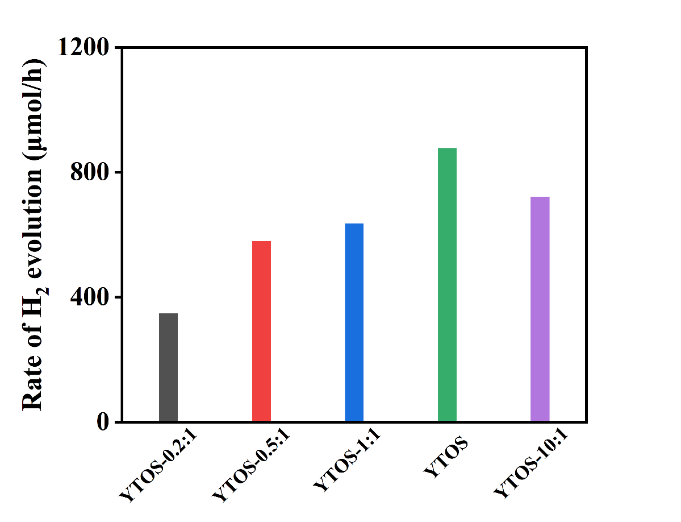


**Figure S9.** H_2_ evolution rates over Pt-modified Y_2_Ti_2_O_5_S_2_ samples. Reaction conditions: 200 mg of photocatalyst loaded with 1 wt% Pt via the impregnation method, 300 W Xe lamp equipped with a cold mirror 1 (CM 1) and a cutoff filter L42 (λ ≥ 420 nm) as the light source, 20 mM Na_2_S-Na_2_SO_3_ solution, 150 mL water, argon at a background pressure of approximately 7.4 kPa.

**
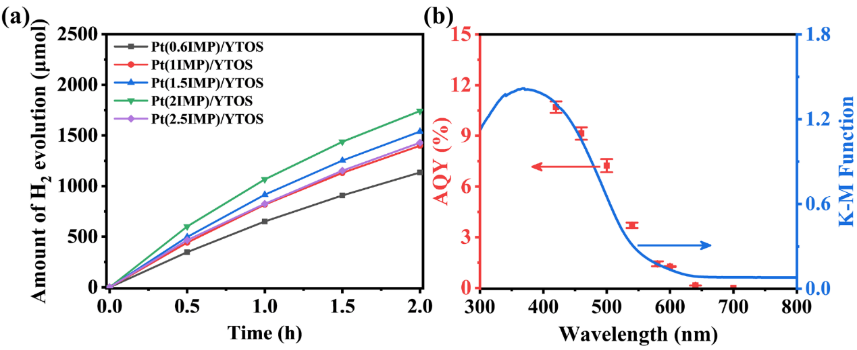
**

**Figure S10.** (a) Effect of amount of the Pt cocatalyst on photocatalytic H_2_ evolution activity. (b) AQY values during H_2_ evolution reaction using Pt(2IMP)/YTOS as function of incident light wavelength. Reaction conditions: 200 mg photocatalyst for (a) and 100 mg photocatalyst for (b), 300 W Xe lamp equipped with cold mirror 1 (CM 1) and cutoff filter L42 (λ ≥ 420 nm) as light source, 20 mM Na_2_S-Na_2_SO_3_ solution, 150 mL water for (a) and 50 ml water for (b), under argon at background pressure of approximately 7.4 kPa. Error bars indicate standard deviations based on three replicate measurements.

**
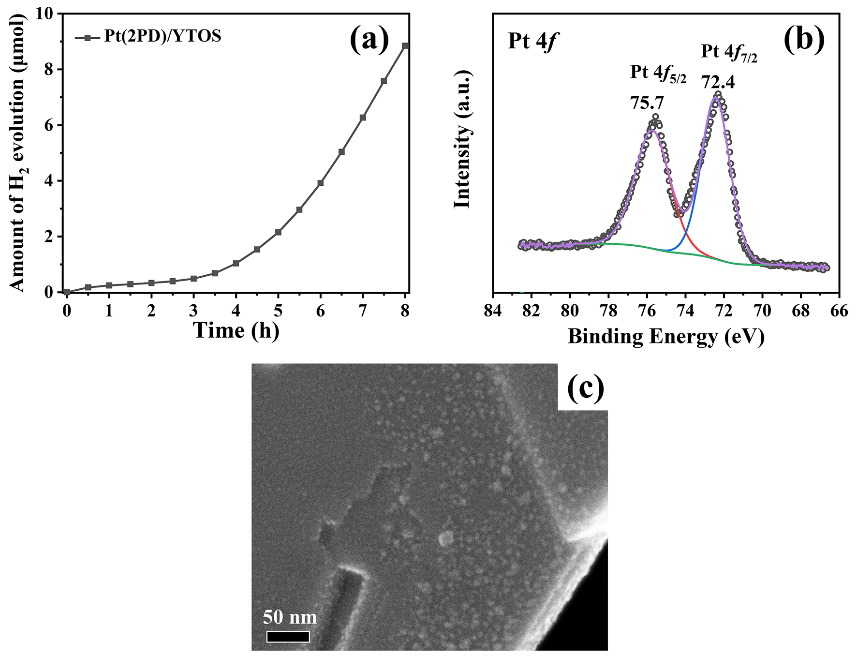
**

**Figure S11.** (a) H_2_ evolution over time during photodeposition of Pt (2 wt%) on YTOS, (b) high-resolution XPS spectrum of Pt 4*f* and (c) SEM image of resulting sample. Reaction conditions: 200 mg photocatalyst, 300 W Xe lamp equipped with a cold mirror 1 (CM 1) and a cutoff filter L42 (*λ* ≥ 420 nm) as the light source, 10 vol% aqueous methanol solution, 150 mL water, under argon at a background pressure of approximately 7.4 kPa.

In the absence of a cocatalyst, the reduction ability of YTOS is extremely weak. The chemical state of Pt(2PD)/YTOS sample was analyzed using X-ray photoelectron spectroscopy (XPS). As shown in Figure S11b, the doublet peaks located at 75.7 and 72.4 eV were assigned to the characteristic splitting of the Pt 4*f*_5/2_ and Pt 4*f*_7/2_ orbitals of Pt^2+^. The main component of nanoparticles loaded on the surfaces of YTOS nanosheets by the photodeposition method is PtO, rather than active metallic Pt. Therefore, the hydrogen evolution activity of Pt(2PD)/YTOS sample was too low.

**
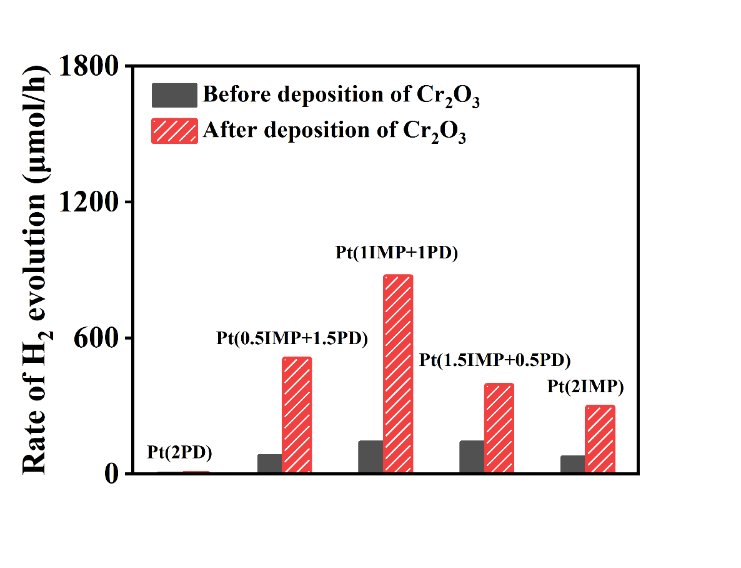
**

**Figure S12.** H_2_ evolution rates over Pt-modified YTOS before and after photodeposition of Cr_2_O_3_ (0.5 wt% Cr). Reaction conditions: 200 mg photocatalyst, 300 W Xe lamp equipped with a cold mirror 1 (CM 1) and a cutoff filter L42 (*λ* ≥ 420 nm) as the light source, 10 vol% aqueous methanol solution, 150 mL water, under argon at a background pressure of approximately 7.4 kPa.


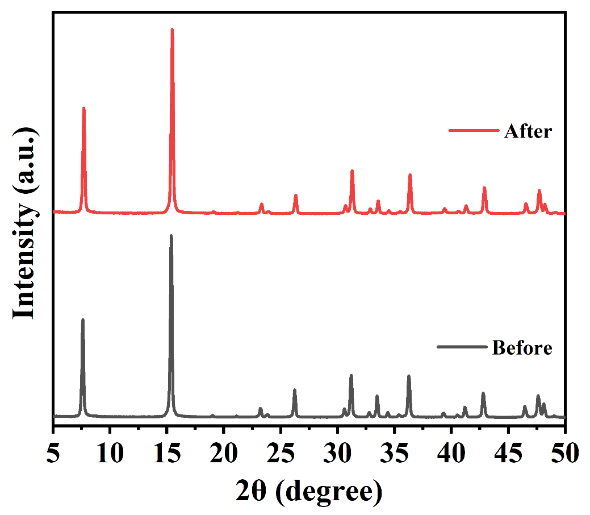


**Figure S13.** XRD patterns for 0.5Cr_2_O_3_-Pt(1IMP+1PD)/YTOS before and after 21 h H_2_ evolution stability test.

**
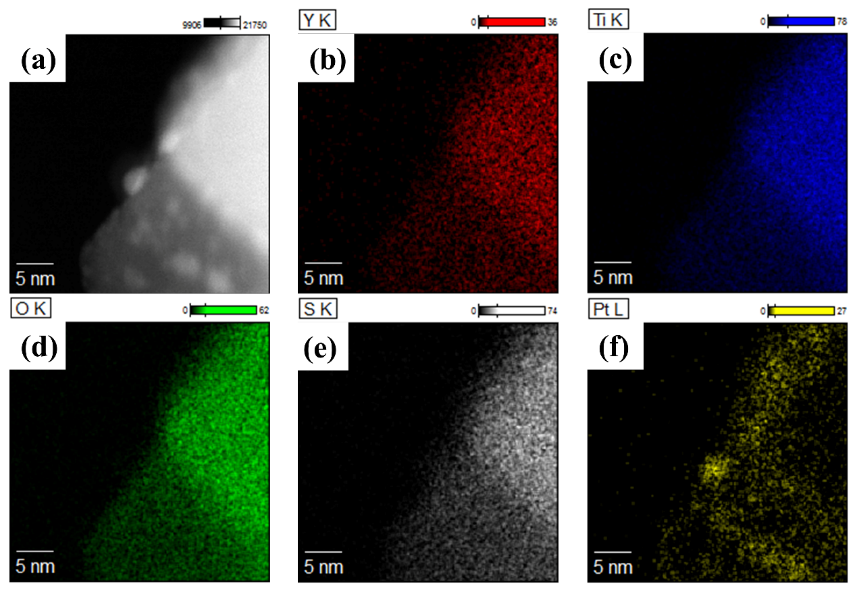
**

**Figure S14.** (a) ADF-STEM image of Pt(1IMP)/YTOS and corresponding STEM-EDS elemental maps showing (b) Y, (c) Ti, (d) O, (e) S, and (f) Pt.

**
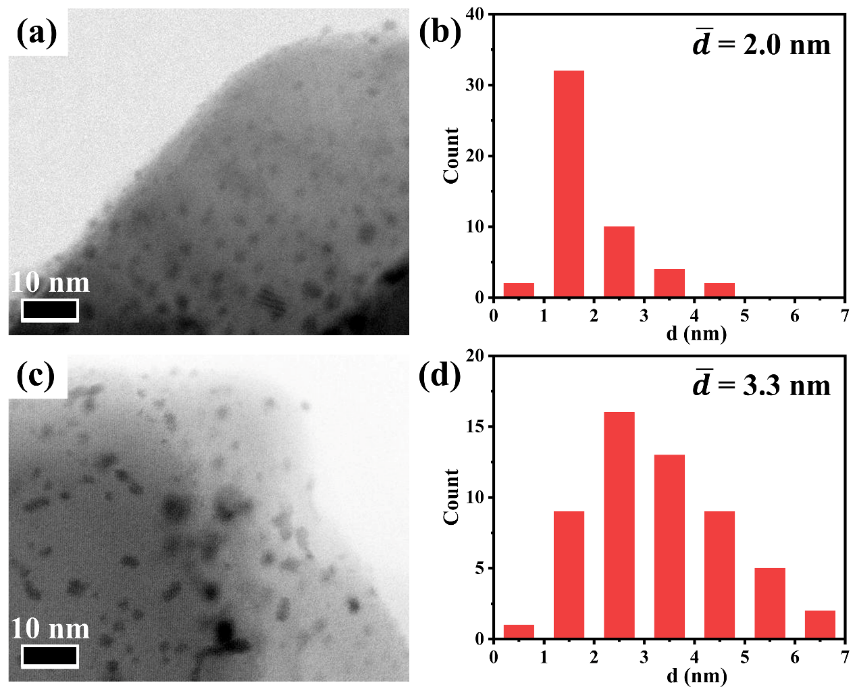
**

**Figure S15.** (a) BF-STEM image of Pt on surface of Pt(1IMP)/YTOS and (b) size distribution of 50 Pt nanoparticles. (c) BF-STEM image of Pt on surface of Pt(1IMP+1PD)/YTOS and (d) size distribution of 50 Pt nanoparticles.

**
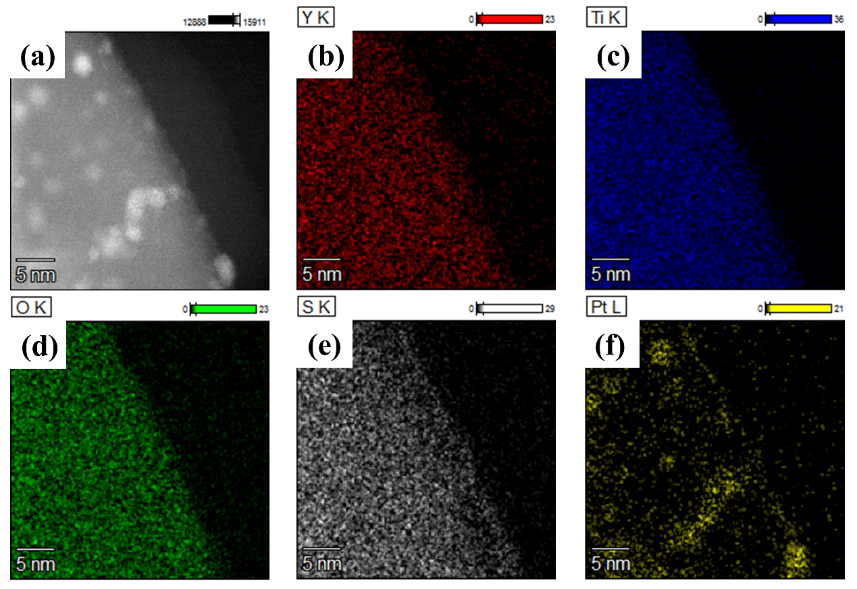
**

**Figure S16.** (a) ADF-STEM image of Pt(1IMP+1PD)/YTOS and corresponding STEM-EDS elemental maps showing (b) Y, (c) Ti, (d) O, (e) S, and (f) Pt.

**
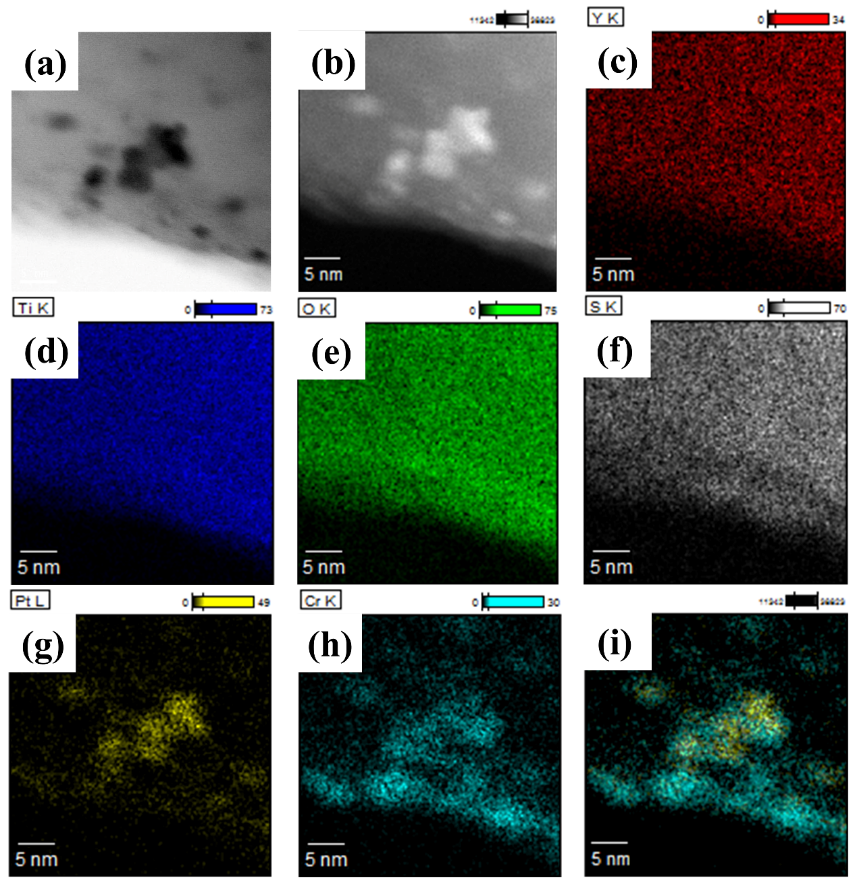
**

**Figure S17.** Observation area 2 for same 0.5Cr_2_O_3_-Pt(1IMP+1PD)/YTOS sample. (a) BF-STEM image and (b) ADF-STEM image of 0.5Cr_2_O_3_-Pt(1IMP+1PD)/YTOS and corresponding STEM-EDS elemental maps showing (c) Y, (d) Ti, (e) O, (f) S, (g) Pt, and (h) Cr. (i) Overlapping map showing distribution of Pt and Cr.

**
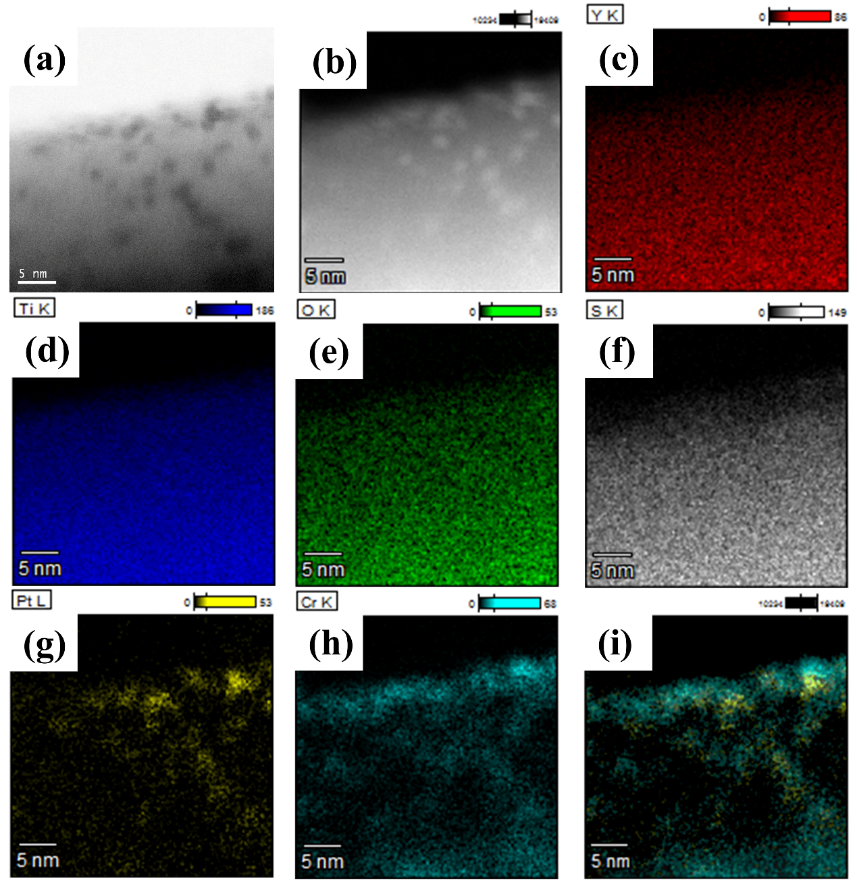
**

**Figure S18.** Observation area 3 for same 0.5Cr_2_O_3_-Pt(1IMP+1PD)/YTOS sample.

(a) BF-STEM image and (b) ADF-STEM image of 0.5Cr_2_O_3_-Pt(1IMP+1PD)/YTOS and corresponding STEM-EDS elemental maps showing (c) Y, (d) Ti, (e) O, (f) S, (g) Pt, and (h) Cr. (i) Overlapping map showing distribution of Pt and Cr.


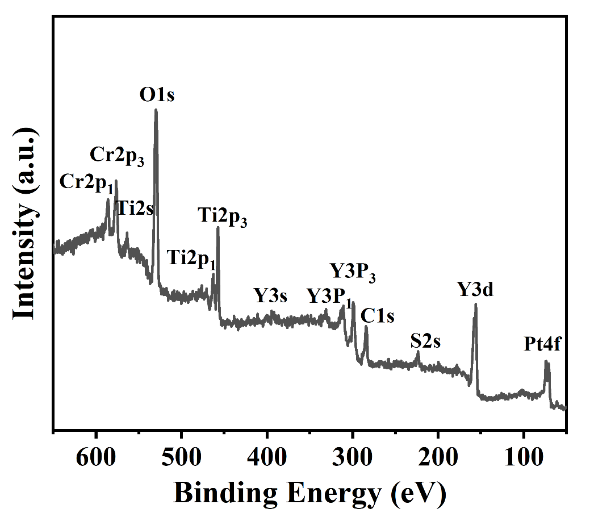


**Figure S19.** (a) Survey XPS data for 0.5Cr_2_O_3_-Pt(1IMP+1PD)/YTOS.


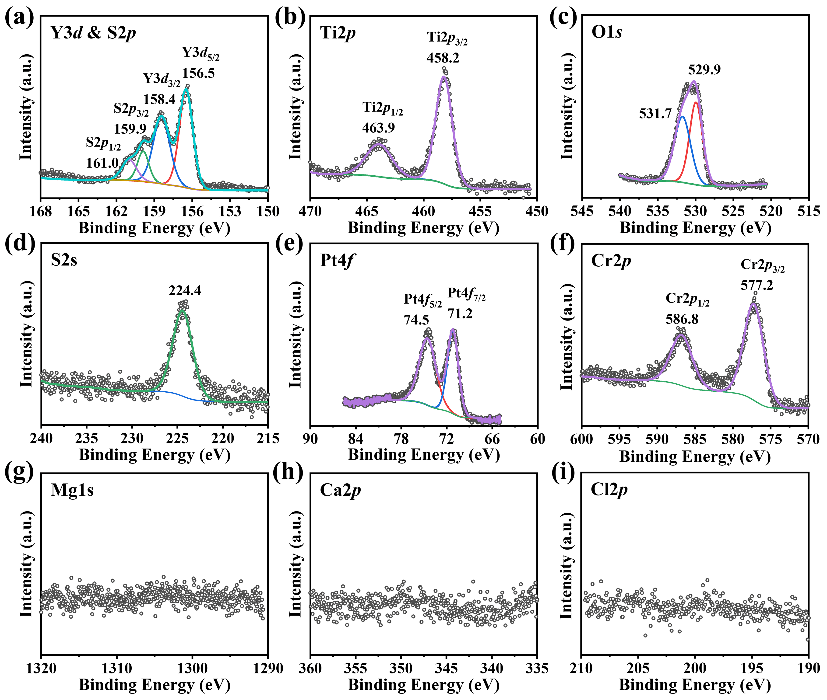


**Figure S20.** High-resolution XPS spectra of (a) Y 3*d* and S 2*p*, (b) Ti 2*p*, (c) O 1*s*, (d) S 2*s*, (e) Pt 4*f*, (f) Cr 2*p*, (g) Mg 1*s*, (h) Ca 2*p*, and (i) Cl 2*p* for 0.5Cr_2_O_3_-Pt(1IMP+1PD)/YTOS.

Figures S19 and S20 provide the survey and high-resolution XPS data obtained from the 0.5Cr_2_O_3_-Pt(1IMP+1PD)/YTOS, respectively. The four peaks appearing in Figure S20a are attributed to S 2*p* and Y 3*d* orbitals. The less intense peaks at 161.0 and 159.9 eV originated from S 2*p*_1/2_ and S 2*p*_3/2_ orbitals associated with S^2-^ while those at 158.4 and 156.5 eV resulted from the Y 3*d*_3/2_ and Y 3*d*_5/2_ orbitals of Y^3+^. The doublet peaks located at 436.9 and 458.2 eV are attributed to the Ti 2*p*_1/2_ and Ti 2*p*_3/2_ orbitals of Ti^4+^ (Figure S20b). The O 1*s* signal was deconvoluted to give peaks at 529.9 and 531.7 eV (Figure S20c). The former originated from lattice oxide ions while the latter can be ascribed to chemisorbed oxygen or hydroxyl groups species. The peak at 224.4 eV was attributed to the S 2*s* orbital associated with S^2-^ in an oxysulfide (Figure S20d). The doublet peaks located at 74.5 and 71.2 eV were assigned to the characteristic splitting of the Pt 4*f*_5/2_ and Pt 4*f*_7/2_ orbitals of metallic Pt (Figure S20e). The presence of this Pt^0^ indicates the complete reduction of the Pt precursor [Pt(IV)Cl_6_]^2-^. The doublet peaks located at 586.8 and 577.2 eV were assigned to the Cr 2*p*_1/2_ and Cr 2p_3/2_ orbitals of Cr^3+^, as demonstrated in Figure S20f. This result confirmed the reduction of K_2_Cr(VI)O_4_ to Cr(III)_2_O_3_ on the surface of the Pt-loaded YTOS. In this case, a Cr_2_O_3_ (shell)/Pt (core) structure was formed during the photodeposition process. It should be noted that a shell made of Cr species would be expected to undergo hydration in water to form Cr(III)O_(1.5−_*_m_*_)_(OH)_2_*_m_*·*x*H_2_O but this shell is herein denoted as Cr_2_O_3_ for brevity.^[S7,S8]^ The impurity signal of MgTiO_3_ was observed in the XRD of YTOS. However, the obvious signal of Mg 1*s* for 0.5Cr_2_O_3_-Pt(1IMP+1PD)/YTOS sample after coating with a layer of Cr_2_O_3_ shell was not observed in XPS analysis (Figure S20g), which may be due to the shallow XPS detection depth. As demonstrated in Figures S20(h, i), no significant peaks characteristic of Ca 2*p* or Cl 2*p* orbitals related to residual flux reagents were observed. These data established that metallic Pt nanoparticles and Cr_2_O_3_ species were deposited on the surface of the YTOS photocatalyst.

**
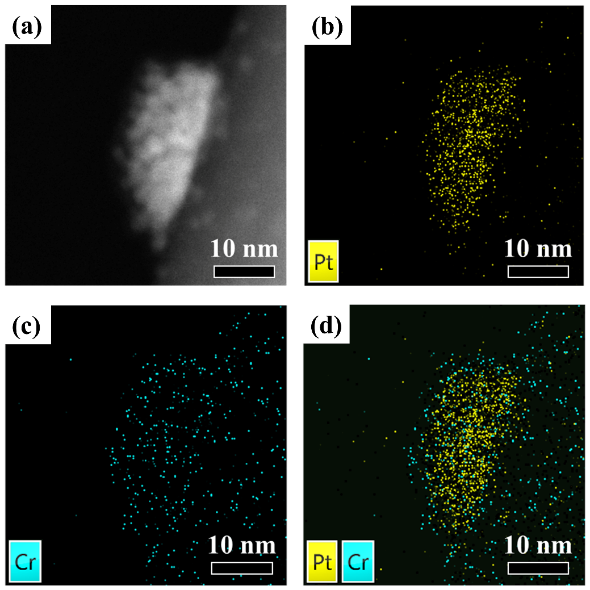
**

**Figure S21.** (a) ADF-STEM image of 0.3Cr_2_O_3_-Pt(1IMP+1PD)/YTOS and corresponding STEM-EDS elemental maps showing (b) Pt and (c) Cr. (d) Overlapping map showing the distributions of Pt and Cr.

**
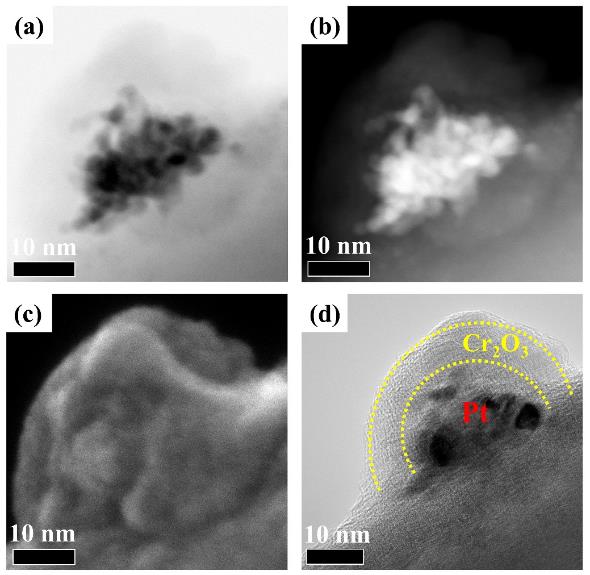
**

**Figure S22.** (a) BF-STEM image, (b) ADF-STEM image, (c) SEM image, and (d) HRTEM image of same 0.8Cr_2_O_3_-Pt(1IMP+1PD)/YTOS sample.


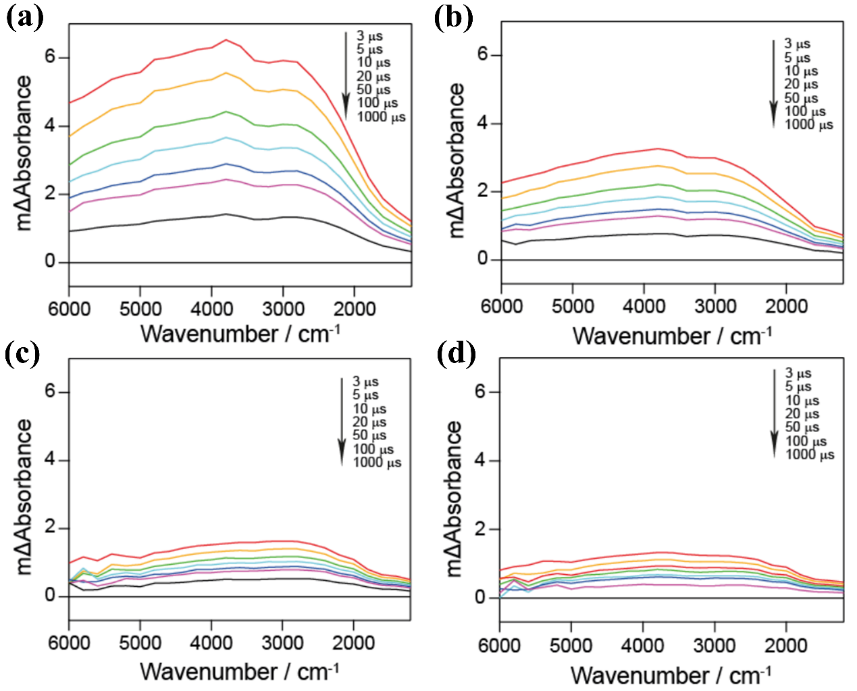


**Figure S23.** TA spectra in mid-IR region acquired over time and corresponding to photoexcited electrons in (a) bare YTOS, (b) Pt(1IMP)/YTOS, (c) Pt(1IMP+1PD)/YTOS, and (d) 0.5Cr_2_O_3_-Pt(1IMP+1PD)/YTOS. Experimental conditions: excitation light source: 470 nm laser pulses; fluence: 0.1 mJ/pulse; frequency: 1 Hz; base pressure: approximately 10^-5^ Torr.

Mid-IR TA spectra acquired from the bare YTOS, Pt(IMP)/YTOS, Pt(IMP+PD)/YTOS and Cr_2_O_3_-Pt(IMP+PD)/YTOS samples at durations of 3 μs to 1 ms are provided in Figures S23(a-d). In this wavelength range, photoexcited electrons are primarily detected. In the case of the bare YTOS, the TA signal intensity drastically decreased after Pt (IMP) and Pt(IMP+PD) were loaded on the YTOS surface, indicating that electrons were captured by the Pt nanoparticles. An additional modification with Cr_2_O_3_ further decreased the signal intensity without changing the shape of the spectra, confirming that the Cr_2_O_3_/Pt core-shell structure promoted electron transfer to the cocatalysts from the YTOS. In addition, as depicted in Figure S23, the TA spectra of bare and cocatalyst loaded YTOS seem to be slightly different. Apparently, there seems to be two absorption peaks near ~3600 and ~2800 cm^-1^ for bare YTOS. But these peaks are not discernible for other samples especially for Pt(1IMP+1PD)/YTOS, and 0.5Cr_2_O_3_-Pt(1IMP+1PD)/YTOS. The dipping feature in the TA spectra observed between 4000 – 2800 cm^-1^, resulting in two apparent absorption peaks, is attributed to the adsorbed water on the surface of YTOS (with peak top ~3400 cm^-1^).^[S9,S10]^ The reason for this may be that a lot of water was adsorbed on the bare YTOS surface and less on Pt(1IMP+1PD)/YTOS and 0.5Cr_2_O_3_-Pt(1IMP+1PD)/YTOS.

**
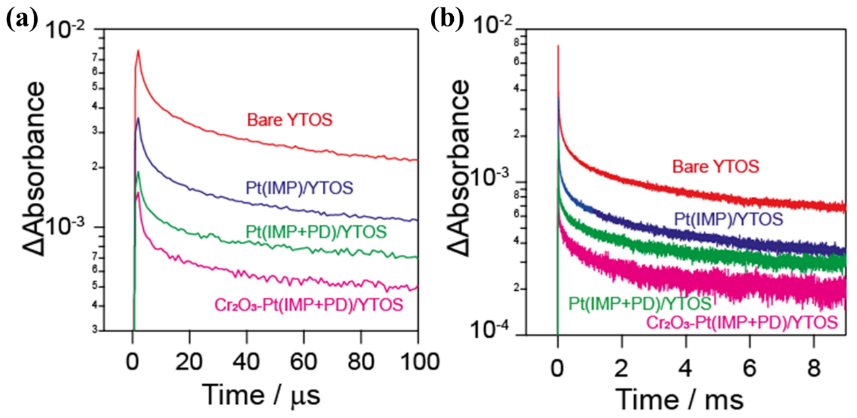
Figure S24.** TA decays corresponding to electron dynamics in bare YTOS, Pt(1IMP)/YTOS, Pt(1IMP+1PD)/YTOS, and 0.5Cr_2_O_3_-Pt(1IMP+1PD)/YTOS based on probing at 5000 cm^−1^ (2000 nm, 0.62 eV) over time span of (a) 0-100 μs and (b) 0-9 ms.

**
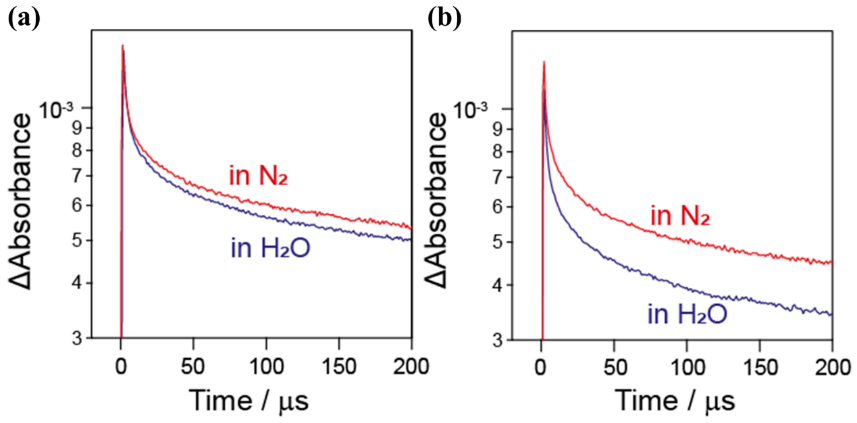
**

**Figure S25.** Changes in electron reactivity following Cr_2_O_3_ loading. TA decays corresponding to electron dynamics in (a) Pt(1IMP+1PD)/YTOS and (b) 0.5Cr_2_O_3_-Pt(1IMP+1PD)/YTOS under N_2_ or water vapor at pressure of 20 Torr based on probing at 2000 cm^−1^ (5000 nm, 0.25 eV) over duration of 0-200 μs.


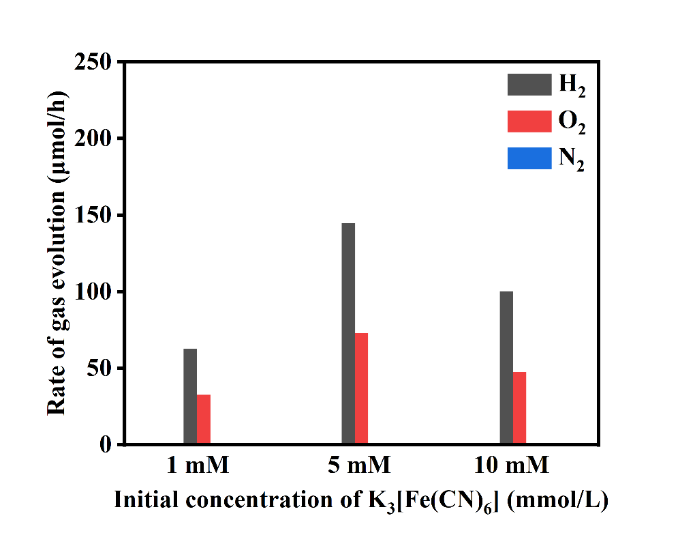


**Figure S26.** Effect of initial concentration of K_3_[Fe(CN)_6_] in solution on photocatalytic Z-scheme water splitting activity. Reaction conditions: 100 mg of the 0.5Cr_2_O_3_-Pt(1IMP+1PD)/YTOS photocatalyst, 200 mg of the Ir-FeCoO*_x_*/BiVO_4_ photocatalyst, 300 W Xe lamp equipped with a cutoff filter (L42; *λ* ≥ 420 nm), 5 mM K_3_[Fe(CN)_6_], 150 mL PBS solution (pH = 6.0), temperature: 285 K. No N_2_ was detected during these Z-scheme water splitting trials.

**
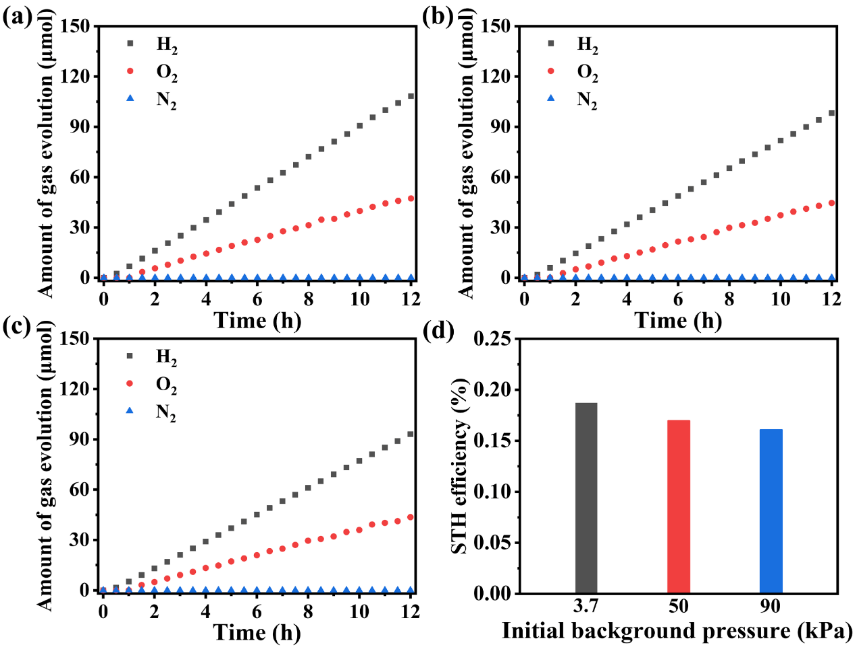
**

**Figure S27.** Gas evolution over time during Z-scheme water splitting reaction using 0.5Cr_2_O_3_-Pt(1IMP+1PD)/YTOS as HEP under simulated sunlight and at argon pressures of (a) 3.7, (b) 50 and (c) 90 kPa. (d) Effect of initial background pressure on STH efficiency of photocatalytic Z-scheme OWS. Reaction conditions: 100 mg of the HEP photocatalyst, 200 mg of the OEP photocatalyst, 300 W Xe lamp with a solar simulator (AM 1.5G, 100 mW cm^-2^), 5 mM K_3_[Fe(CN)_6_], 150 mL PBS solution (pH = 6.0), temperature: 285 K.





**Figure S28.** Comparison of water formation reaction over bare YTOS (red spheres) and Pt(1IMP+1PD)/YTOS (bule triangles) under darkness. For the experiment, an initial stoichiometric mixture of H_2_ and O_2_ gases (~2000 μmol for H_2_ and ~1000 μmol for O_2_) was introduced to the closed circulation system.

**Table S1.** Hydrogen evolution half-reaction activity of the present Y_2_Ti_2_O_5_S_2_ photocatalysts.

| **Photocatalyst** | **Cocatalyst** | **Reaction solution** | **Mass(mg)** | **Gas evolution rates^[a]^ (μmol h^−1^)** | **Efficiency^[b]^** | **Ref.** |
| --- | --- | --- | --- | --- | --- | --- |
| Y_2_Ti_2_O_5_S_2_ | Rh | Na_2_S/Na_2_SO_3_ | 200 | H_2_: ~125 | AQY: ~5.3% at  420 nm | [S11] |
| Sc:Y_2_Ti_2_O_5_S_2_ (KI) | Rh | Na_2_S/Na_2_SO_3_ | 300 | H_2_: ~445 | N/A | [S12] |
| Flux-assisted Y_2_Ti_2_O_5_S_2_ | Rh | Na_2_S/Na_2_SO_3_ | 200 | H_2_: ~387 | AQY: ~5.9% at  420 nm | [S5] |
| Flux-assisted Y_2_Ti_2_O_5_S_2_ | Pt@Au-IrO_2_ | Na_2_S/Na_2_SO_3_ | 50 | H_2_: ~978 | AQY: ~18.2% at 420 nm | [S13] |
| Flux-assisted Y_2_Ti_2_O_5_S_2_ | Pt | Na_2_S/Na_2_SO_3_ | 200 | H_2_: ~1066 | AQY: ~10.7% at 420 nm | **This work** |
| Flux-assisted Y_2_Ti_2_O_5_S_2_ | Cr_2_O_3_/Pt | Na_2_S/Na_2_SO_3_ | 200 | H_2_: ~430 | AQY: ~4.0% at 420 nm | **This work** |
| Flux-assisted Y_2_Ti_2_O_5_S_2_ | Cr_2_O_3_/Pt | Methanol solution | 200 | H_2_: ~765 | AQY: ~7.2% at 420 nm | **This work** |

^[a]^ Light source: 300 W Xe lamp; *λ* ≥ 420 nm.

^[b]^ AQY: Apparent quantum yield; N/A: not available.

**Table S2.** Comparison of Z-scheme water splitting systems responsive to wide range of wavelengths.^[a]^

| **HEP** | **OEP** | **Electron**  **Mediator** | **Reaction solution** | **Efficiency^[f]^** | **Ref.** |
| --- | --- | --- | --- | --- | --- |
| Ru/SrTiO_3_:Rh  (＜520 nm) | BiVO_4_  (＜520 nm) | None | H_2_SO_4_ solution  pH = 3.5 | AQY: 1.7% at 420 nm;  STH: 0.12% | [S14] |
| Ru/SrTiO_3_:La,Rh  (＜520 nm) | BiVO_4_:Mo  (＜520 nm) | Au, C, or ITO^[c]^ | H_2_O | STH: 1.1% (Au); STH: 1.2% (C); STH: 0.4% (ITO) | [S15-S17] |
| Pt/TiO_2_/CdS/ (ZnSe)_0.5_(CuGa_2.5_Se_4.25_)_0.5_  (＜725 nm) | BiVO_4_:Mo  (＜520 nm) | Au layer | H_2_O | AQY 1.5% at 420 nm | [S18] |
| Cr_2_O_3_/Rh/LTCA:Mg,Al^[b]^  (< 700 nm) | CoO_x_/BiVO_4_:Mo  (＜520 nm) | Au layer | H_2_O | AQY 16.3% at 420 nm;  STH: 0.67% | [S19] |
| RhCrO_x_/LaMg_1/3_Cu_2/3_O_2_N  (< 600 nm) | CoO_x_/BiVO_4_:Mo  (＜520 nm) | Au and RGO^[d]^ | H_2_O | AQY 0.25% at 418 nm;  STH: 0.0035% | [S20] |
| Pt/(CuGa)_0.5_ZnS_2_  (＜560 nm) | CoO_x_/BiVO_4_  (＜520 nm) | RGO^[d]^ | H_2_O | AQY: 0.80% at 440 nm;  STH: 0.024% | [S21] |
| Cr_2_O_3_/Pt/IrO_2_/Sm_2_Ti_2_S_2_O_5_  (＜650 nm) | CoO_x_/BiVO_4_  (＜520 nm) | RGO^[d]^ | H_2_O | AQY 7.0% at 420 nm;  STH: 0.22% | [S3] |
| Cr_2_O_3_/Pt/IrO_2_/Sm_2_Ti_2_S_2_O_5_  (＜650 nm) | CoO_x_/BiVO_4_  (＜520 nm) | CNTs^[e]^ | H_2_O | AQY 5.1% at 420 nm;  STH: 0.15% | [S4] |
| Ru/SrTiO_3_:La,Rh  (＜520 nm) | CoO_x_/Ta_3_N_5_  (＜600 nm) | Ir | H_2_SO_4_ solution  pH = 3.9 | AQY: 1.1% at 420 nm;  STH: 0.037% | [S22] |
| ZnRh_2_O_4_  (＜1030 nm) | Bi_4_V_2_O_11_  (＜750 nm) | Ag | H_2_O | AQY: 0.003% at 740 nm | [S23] |
| Pt/SrTiO_3_:Rh  (＜520 nm) | BiVO_4_  (＜520 nm) | [Co(bpy)_3_]^3+/2+^  or [Co(phen)_3_]^3+/2+^ | H_2_O | AQY: 2.1% at 420 nm | [S24] |
| RhyCr_2–y_O_3_-ZrO_2_/TaON  (<520 nm) or Rh_y_Cr_2–y_O_3_-  MgTa_2_O_6–x_N_y_/TaON  (< 570 nm) | Ir-FeCoO_x_/BiVO_4_  (＜520 nm) | [Fe(CN)_6_]^3−^/^4−^ | Buffer solution  pH = 6.0 | AQY 12.3% at 419 nm;  STH: 0.6% | [S1] |
| Ru/SrTiO_3_:Rh  (＜520 nm) | BiVO_4_  (＜520 nm) | Fe^3+^/Fe^2+^ | H_2_SO_4_ solution  pH = 2.4 | AQY: 4.2% at 420 nm;  STH: 0.1% | [S25] |
| Rh_2–y_Cr_y_O_3_@ Au–IrO_2_/Y_2_Ti_2_O_5_S_2_  (＜650 nm) | CoO_x_/BiVO_4_  (＜520 nm) | [Co(bpy)_3_]^2+/3+^ | H_2_O | AQY 4.14% at 420 nm; STH: 0.15% | [S13] |
| Cr_2_O_3_/Pt/Y_2_Ti_2_O_5_S_2_  (＜650 nm) | Ir/FeCoO_x_/BiVO_4_  (＜520 nm) | [Fe(CN)_6_]^3−^/^4−^ | Phosphate buffer solution (pH 6.0) | AQY 4.5% at 420 nm;  STH: 0.19% | **This work** |

^[a]^ Defined as Z-scheme water splitting system with both the HEP and OEP having an absorption edge wavelength longer than 520 nm.

^[b]^ LTCA: La_5_Ti_2_Cu_0.9_Ag_0.1_S_5_O_7_.

^[c]^ ITO: indium tin oxide.

^[d]^ RGO: reduced graphene oxide.

^[e]^ CNTs: carbon nanotubes.

^[f]^ AQY: Apparent quantum yield and STH: Solar-to-hydrogen energy conversion efficiency (the higher value in the report was taken).

**References**

[S1] Y. Qi, J. Zhang, Y. Kong, Y. Zhao, S. Chen, D. Li, W. Liu, Y. Chen, T. Xie, J. Cui, C. Li, K. Domen, F. Zhang, *Nat. Comm.* **2022**, *13*, 484.

[S2] L. Lin, V. Polliotto, J. J. M. Vequizo, X. Tao, X. Liang, Y. Ma, T. Hisatomi, T. Takata, K. Domen, *ChemPhotoChem* **2022**, *6*, e202200209.

[S3] L. Lin, Y. Ma, J. J. M. Vequizo, M. Nakabayashi, C. Gu, X. Tao, H. Yoshida, Y. Pihosh, Y. Nishina, A. Yamakata, N. Shibata, T. Hisatomi, T. Takata, K. Domen, *Nat. Commun.* **2024**, *15*, 397.

[S4] L. Lin, Y. Ma, N. Zettsu, J. J. M. Vequizo, C. Gu, A. Yamakata, T. Hisatomi, T. Takata, K. Domen, *J. Am. Chem. Soc.* **2024**, *146*, 14829.

[S5] L. Lin, P. Kaewdee, V. Nandal, R. Shoji, H. Matsuzaki, K. Seki, M. Nakabayashi, N. Shibata, X. Tao, X. Liang, Y. Ma, T. Hisatomi, T. Takata, K. Domen, *Angew. Chem. Int. Ed.* **2023**, *62*, e202310607.

[S6] F. Zuo, L. Wang, T. Wu, Z. Zhang, D. Borchardt, P. Feng, *J. Am. Chem. Soc.* **2010**, *132*, 11856.

[S7] M. Yoshida, K. Takanabe, K. Maeda, A. Ishikawa, J. Kubota, Y. Sakata, Y. Ikezawa, K. Domen, *J. Phys. Chem. C* **2009**, *113*, 10151.

[S8] T. Takata, J. Jiang, Y. Sakata, M. Nakabayashi, N. Shibata, V. Nandal, K. Seki, T. Hisatomi, K. Domen, *Nature* **2020**, *581*, 411.

[S9] A. Yamakata, M. Osawa, *J. Electroanal. Chem.* **2017**, *800*, 19.

[S10] A. Litke, E. J. M. Hensen, J. P. Hofmann. *J. Phys. Chem. C* **2017**, *121*, 10153.

[S11] Q. Wang, M. Nakabayashi, T. Hisatomi, S. Sun, S. Akiyama, Z. Wang, Z. Pan, X. Xiao, T. Watanabe, T. Yamada, N. Shibata, T. Takata, K. Domen, *Nat. Mater.* **2019**, *18*, 827.

[S12] H. Yoshida, Z. Pan, R. Shoji, V. Nandal, H. Matsuzaki, K. Seki, T. Hisatomi, K. Domen, *J. Mater. Chem. A* **2022**, *10*, 24552.

[S13] J. Zhang, K. Liu, B. Zhang, J. Zhang, M. Liu, Y. Xu, K. Shi, H. Wang, Z. Zhang, P. Zhou, G. Ma, *J. Am. Chem. Soc.* **2024**, *146*, 4068.

[S14] Y. Sasaki, H. Nemoto, K. Saito, A. Kudo, *J. Phys. Chem. C* **2009**, *113*, 17536.

[S15] Q. Wang, T. Hisatomi, Q. Jia, H. Tokudome, M. Zhong, C. Wang, Z. Pan, T. Takata, M. Nakabayashi, N. Shibata, Y. Li, I. D. Sharp, A. Kudo, T. Yamada, K. Domen, *Nat. Mater.* **2016**, *15*, 611.

[S16] Q. Wang, T. Hisatomi, Y. Suzuki, Z. Pan, J. Seo, M. Katayama, T. Minegishi, H. Nishiyama, T. Takata, K. Seki, A. Kudo, T. Yamada, K. Domen, *J. Am. Chem. Soc.* **2017**, *139*, 1675.

[S17] Q. Wang, S. Okunaka, H. Tokudome, T. Hisatomi, M. Nakabayashi, N. Shibata, T. Yamada, K. Domen, *Joule* **2018**, *2*, 2667.

[S18] S. Chen, J. J. M. Vequizo, Z. Pan, T. Hisatomi, M. Nakabayashi, L. Lin, Z. Wang, K. Kato, A. Yamakata, N. Shibata, T. Takata, T. Yamada, K. Domen, *J. Am. Chem. Soc.* **2021**, *143*, 10633.

[S19] S. Nandy, T. Hisatomi, M. Nakabayashi, H. Li, X. Wang, N. Shibata, T. Takata, K. Domen, *Joule* **2023**, *7*, 1641.

[S20] Z. Pan, T. Hisatomi, Q. Wang, S. Chen, A. Iwase, M. Nakabayashi, N. Shibata, T. Takata, M. Katayama, T. Minegishi, A. Kudo, K. Domen, *Adv. Funct. Mater.* **2016**, *26*, 7011.

[S21] S. Yoshino, A. Iwase, Y. H. Ng, R. Amal, A. Kudo, *ACS Appl. Energy Mater.* **2020**, *3*, 5684.

[S22] Q. Wang, T. Hisatomi, S. S. K. Ma, Y. Li, K. Domen, *Chem. Mater.* **2014**, *26*, 4144.

[S23] R. Kobayashi, T. Takashima, S. Tanigawa, S. Takeuchi, B. Ohtani, H. A, Irie, *Phys. Chem. Chem. Phys.* **2016**, *18*, 27754.

[S24] Y. Sasaki, H. Kato, A. Kudo, *J. Am. Chem. Soc.* **2013**, *135*, 5441.

[S25] H. Kato, Y. Sasaki, N. Shirakura, A. Kudo, *J. Mater. Chem. A* **2013**, *1*, 12327.
